# Alcohol intake and associated risk of major cardiovascular outcomes in women compared with men: a systematic review and meta-analysis of prospective observational studies

**DOI:** 10.1186/s12889-015-2081-y

**Published:** 2015-08-12

**Authors:** Yan-Ling Zheng, Feng Lian, Qian Shi, Chi Zhang, Yi-Wei Chen, Yu-Hao Zhou, Jia He

**Affiliations:** Department of Teaching Affairs and Scientific Research, Shandong Medical College, Jinan, Shandong China; Department of Ultrasonography, Shanghai Seventh People’s Hospital, Shanghai, China; Department of Neurosurgery, Shanghai Seventh People’s Hospital, Shanghai, China; Department of Rehabilitation Institute, Shanghai Seventh People’s Hospital, Shanghai, China; Department of Health Statistics, Second Military Medical University, Shanghai, China

**Keywords:** Alcohol intake, Coronary disease, Total mortality, Stroke, Meta-analysis

## Abstract

**Background:**

The prevalence of alcohol intake is increasing among women in some populations. Alcohol consumption plays an important role in the risk of major cardiovascular outcomes and total mortality. Here, we conducted a meta-analysis to estimate the association between alcohol intake and major cardiovascular outcomes or total mortality in women compared with men.

**Methods:**

We searched the PubMed, Embase, and the Cochrane Library databases for relevant articles published prior to June 2014. Among these potential included prospective studies, the different dose categories of alcohol intake were compared with the lowest alcohol intake or non-drinkers between women and men for the outcomes of major cardiovascular or total mortality.

**Results:**

We included 23 prospective studies (18 cohorts) reporting data on 489,696 individuals. The summary relative risk ratio (RRR; female to male) for total mortality was significantly increased with moderate alcohol intake compared with the lowest alcohol intake (RRR, 1.10; 95 % confidence interval [CI]: 1.00–1.21; *P* = 0.047); no such significance was observed with other levels of alcohol intake (low intake: RRR, 1.07; 95 % CI: 0.98–1.17; *P* = 0.143; heavy intake: RRR, 1.09; 95 % CI: 0.99–1.21; *P* = 0.084). There was no evidence of a sex difference in the relative risk for coronary disease, cardiac death, stroke, or ischemic stroke between participants with low to heavy alcohol intake compared with those who never consumed alcohol or had the lowest alcohol intake.

**Conclusions:**

Women with moderate to heavy alcohol intake had a significantly increased risk of total mortality compared with men in multiple subpopulations. Control of alcohol intake should be considered for women, particularly for young women who may be susceptible to binge drinking.

**Electronic supplementary material:**

The online version of this article (doi:10.1186/s12889-015-2081-y) contains supplementary material, which is available to authorized users.

## Background

Alcohol is a commonly consumed beverage in many populations, and contributes both favorably and adversely to disease morbidity and mortality [1]. A large number of cohort studies have shown that light-to-moderate alcohol intake is associated with a decreased risk of cardiovascular disease and ischemic stroke, and that heavy intake is associated with an increased risk of hemorrhagic stroke among men [2–7]. Previous studies [8, 9] have indicated that women with light-to-moderate alcohol intake have a significantly lower relative risk of cardiovascular disease compared to male drinkers, furthermore, there are some debate as to whether this sex difference is true for the association between alcohol intake and major cardiovascular outcomes or total mortality.

In 1998, the Multiethnic Prospective Cohort (MPC) study [10] indicated that women with heavy alcohol intake had a 203 % greater stroke risk and 17 % greater total mortality risk compared to men. However, in the Melbourne Collaborative Cohort Study [9], the risk of coronary disease was found to be 67 % lower in women with heavy alcohol intake compared with men. Similarly, the risk of coronary disease was found to be 55 % lower in women with moderate alcohol intake compared to men in the Danish National Cohort Study (DANCOS) [8]. The reasons for this variation in the sex-specific association between alcohol intake and subsequent major cardiovascular outcomes could be the different study designs, the classification of alcohol type, and the different adjusted confounding factors.

At present, it is unclear whether women who consume alcohol are at a greater risk or a benefit of major cardiovascular outcomes than men. Herein, we conducted a systematic review and meta-analysis of the available prospective observational studies to evaluate those effects of alcohol intake on the subsequent risk of major cardiovascular outcomes or total mortality in women compared with men.

## Methods

### Data sources, search strategy, and selection criteria

This review was conducted and was reported according to the Preferred Reporting Items for Systematic Reviews and Meta-Analysis Statement issued in 2009 [11]. Any prospective observational study that evaluated the association between alcohol intake and subsequent major cardiovascular outcomes or total mortality risk in men and women was eligible for inclusion in our study, and no restrictions were placed on language or publication status. Relevant studies were identified using the following procedure:

1. Electronic searches: We searched the PubMed, Embase, Ovid, and the Cochrane Library databases for articles published through June 2014. Both medical subject headings and free-language terms of *“ethanol” or “alcohol” or “alcoholic beverages” or “drinking behaviour” or “alcohol drinking” AND (“stroke” or “cardiovascular diseases” or “myocardial infarction” or “myocardial ischemia” or “coronary artery disease” or “heart infarction”) AND “men” AND “women” AND (“cohort” or “prospective” or “nested case–control”)*were used as search terms (Additional file 1).
2. Other sources: Meeting abstracts, references of meta-analyses, and reviews already published on related topics were examined. Authors were contacted for essential information regarding publications that were not available in full. The medical subject heading, methods, population, study design, exposure, and outcome variables of these articles were used to identify relevant studies.

The literature search, data extraction, and quality assessment were independently undertaken by two investigators (YHZ and CZ) using a standardized approach. Any inconsistencies between these investigators were identified by the primary investigator (JH) and resolved by consensus. We restricted our study to prospective observational studies that were less likely to be subject to confounding variables or bias compared to traditional case control studies [12]. A study would be eligible for inclusion in our meta-analysis if the following criteria were met: (1) the study was a prospective observational study (prospective cohort study or nested case control study); (2) the study investigated the association between alcohol intake and the risk of major cardiovascular outcomes or total mortality in men and women separately; and (3) the authors reported effect estimate (risk ratio [RR], odds ratio [OR], or hazard ratio [HR]) and 95 % confidence intervals (CIs) on cardiovascular outcomes (coronary disease, total mortality, cardiac death, stroke, and ischemic stroke) for comparisons of different dosage of alcohol intake with the lowest alcohol intake or non-drinking.

### Data collection and quality assessment

The information collected the included group’s name, country, study design, sample size for men and women, age at baseline, percentage of sample size for different alcohol intake categories, follow-up duration, and covariates in the fully adjusted model. We also extracted the effect estimate and its 95 % CIs. For studies that reported several multivariable adjusted RRs, we selected the effect estimate that was maximally adjusted for potential confounders.

The Newcastle-Ottawa Scale (NOS) has been partially validated for evaluating the quality of observational studies, was employed to evaluate methodological quality [13, 14]. The NOS is based on the following three subscales: selection (four items), comparability (one item), and outcome (three items). A “star system” (range, 0–9) has been developed for assessment [13]. The data extraction and quality assessment were conducted independently by two authors (YHZ and FL). Referring to the original studies, information was examined and adjudicated independently by an additional author (JH).

### Statistical analysis

We examined the relationship between alcohol intake and the risk of major cardiovascular outcomes or total mortality in women compared with men based on the effect estimate (RR, OR, or HR) and its 95 % CI in each study. For every study, sex-specific RRs and 95 % CIs were used to estimate the female-to-male ratio of RRs (relative risk ratio [RRR]) and its 95 % CIs [15]. First, we used a random effects model to calculate summary RRs and 95 % CIs for different exposure categories versus the lowest alcohol intake in men and women separately. Next, both fixed-effect and random-effect models were used to evaluate the pooled RRR for the comparison of different exposure categories versus the lowest alcohol intake in women compared with men; the results from the random-effect model which assumed that the true underlying effect varied among included trials, were presented here [16, 17].

Heterogeneity between studies was investigated using the Q statistic, and we considered *P*-values of < 0.10 as indication of significant heterogeneity [18–20]. Subgroup analyses were conducted for coronary disease or total mortality based on the country, sample size, physical activity, serum cholesterol, hypertension, diabetes, follow-up duration, and the study quality.

We also performed a sensitivity analysis by removing a specific study from the meta-analysis [21]. Several methods were used to check for potential publication bias. Visual inspections of funnel plots for coronary disease and total mortality were conducted. The Egger [22] and Begg tests [23] were also used to statistically assess publication bias for coronary disease and total mortality. All reported P-values were 2-sided and *P*-values of <0.05 were considered statistically significant for all included studies. Statistical analyses were performed using STATA software (version 12.0; Stata Corporation, College Station, TX, USA).

## Results

### Studies and patient characteristics

**Fig. 1:**
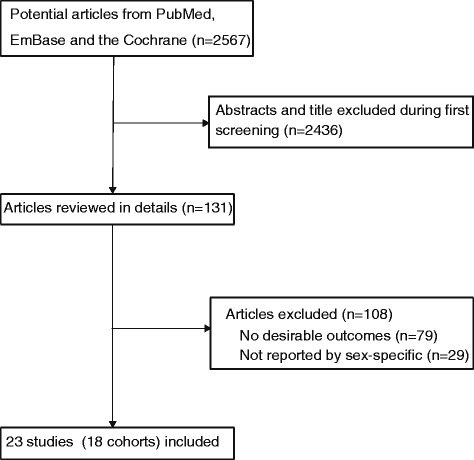
Flow diagram of the literature search and studies selection process

The results of the study selection process were shown in Fig. 1. We identified 2,567 articles in our initial electronic search, of which 2,436 were excluded because they were duplicate or irrelevant articles. A total of 131 potentially eligible studies were selected. After detailed evaluations, 23 prospective studies including 18 cohorts were selected for the final meta-analysis [8–10, 24–43]. A manual search of the reference lists within these studies did not yield any new eligible studies. The general characteristics of the included studies were presented in Tables 1 and Additional file 2: Table S1.

**Table 1:** Baseline characteristic of studies included

Study	Country	Sex	Study design	Sample size	Age at baseline	Ex-drinker (%)	Light alcohol (%)	Moderate alcohol (%)	Heavy alcohol (%)	Follow-up (year)	Main outcomes	NOS score
Framingham [24]	US	Men	Cohort	3871	>50	19.9	30.0	14.7	35.4	10.0	Ischemic stroke	8
		Women	Cohort	5300	>50	37.9	39.1	9.6	13.4			
DANCOS [8]	Denmark	Men	Cohort	12994	19–75	14.4	27.8	41.5	16.3	6.9	Coronary disease, total mortality	7
		Women	Cohort	13794	20–79	27.9	39.8	28.8	3.8			
EPOZ [25]	Netherlands	Men	Cohort	760	-	6.7	17.6	38.6	37.1	10.0	Cardiac death, total mortality	6
		Women	Cohort	860	-	18.4	29.3	34.9	17.4			
MPC [10]	US	Men	Cohort	13870	>30	60.9	14.4	10.0	12.8	20.0	Coronary disease, stroke, total mortality	7
		Women	Cohort	13808	>30	85.6	8.4	3.2	2.2			
NHEFS [26]	US	Men	Cohort	768	46.3	17.8	63.8	7.0	11.5	17.0	Total mortality	5
		Women	Cohort	1286	45.7	36.7	56.5	3.2	3.7			
Whitehall II [27]	UK	Men	Cohort	6840	35–55	13.0	45.6	12.6	28.7	11.0	Coronary disease, total mortality	7
		Women	Cohort	3374	35–55	29.1	54.8	11.4	4.7			
MONICA/KORA-Augsburg [28]	Germany	Men	Cohort	1345	35–64	7.4	19.7	23.8	34.5	10.1	Total mortality	7
		Women	Cohort	1365	35–64	28.3	30.0	15.8	-			
MCCS [9]	Australia	Men	Cohort	15156	40–69	13.2	39.9	20.8	16.1	11.4	Coronary disease	8
		Women	Cohort	23044	40–69	38.3	37.3	11.0	-			
CCHS [29]	Denmark	Men	Cohort	5272	>20	15.3	-	51.0	33.7	20.0	Coronary disease, total mortality	9
		Women	Cohort	6642	>20	42.8	-	50.4	6.8			
HPFS and NHS [30]	US	Men	Cohort	43685	54	-	-	-	-	18-20	Total stroke, ischemic stroke	6
		Women	Cohort	71243	50	-	-	-	-			
LWCS [31]	US	Men	Cohort	4852	74	21.6	24.9	27.0	26.5	12.0	Cardiac death	7
		Women	Cohort	8444	73	27.7	35.4	21.3	15.7			
DDCHS [32, 33]	Denmark	Men	Cohort	26035	50–65	4.9	52.3	11.9	30.9	5.7	Coronary disease	8
		Women	Cohort	29427	50–65	15.8	66.0	9.2	9.0			
JACC [34–36]	Japan	Men	Cohort	34776	40–79	22.5	17.6	23.2	29.9	14.2	Cardiac death, total mortality, stroke, ischemic stroke	9
		Women	Cohort	48906	40–79	83.5	12.0	2.1	0.7			
EPIC-Nutrition-Heidelberg [37]	Germany	Men	Cohort	11062	42–62	30.0	31.0	30.0	9.0	12.0	Total stroke	7
		Women	Cohort	12865	37–62	60.0	22.0	13.0	5.0			
EPIC-Potsdam [38]	Germany	Men	Nested case-cohort	815	35–65	3.2	33.4	24.5	38.9	8.2	Coronary disease, stroke	6
		Women	Nested case-cohort	1360	35–65	2.6	81.9	14.1	7.4			
EPIC-Spanish [39]	Spain	Men	Cohort	15630	29–69	4.0	13.5	32.6	41.3	10.0	Coronary disease	7
		Women	Cohort	25808	29–69	35.1	33.8	21.3	3.0			
Lifestyle and health study [40, 41]	Netherlands	Men	Nested case-cohort	1961	45–70	-	-	-	-	5.0	Coronary disease, total mortality	5
		Women	Nested case-cohort	1713	45–70	-	-	-	-			
NHIS [42, 43]	US	Men	Cohort	10998	>18	-	23.0	30.0	47.0	14.0	Cardiac death, total mortality	8
		Women	Cohort	9767	>18	-	33.3	36.6	31.1			

Of the 18 included cohorts, reporting data on 489,696 individuals, 16 cohorts (20 studies) had been examined using a prospective cohort study design [8–10, 24–37, 39, 42, 43], and the remaining two cohorts (three studies) had been examined by using a prospective nested case control study design [38, 40, 41]. The follow-up period for participants was 5.0–20.0 years, and 1,620–114,928 individuals were included in each study. A total of six cohorts (seven studies) were conducted in the US [10, 24, 26, 30, 31, 42, 43], ten (13 studies) were conducted in Europe [8, 25, 27–29, 32, 33, 35, 37–41], and two (three studies) were conducted in other countries [9, 34, 36]. Study quality was assessed using the NOS. Overall, two cohorts had a score of 9 [29, 34–36], four cohorts had a score of 8 [9, 24, 32, 33, 42, 43], seven cohorts had a score of 7 [8, 10, 27, 28, 31, 37, 39], three cohorts had a score of 6 [25, 30, 38], and the remaining two cohorts had a score of 5 [26, 40, 41].

### Coronary disease

A total of nine cohorts (11 studies) reported an association between alcohol intake and the risk of coronary disease [8–10, 27, 29, 32, 33, 38–41]. The summary RR of the associated between alcohol intake and coronary disease in men and women, were separately listed in Table 2. The pooled RRR (female to male) of low alcohol intake (<15 g/day) versus the lowest alcohol or no alcohol intake was 1.01 (95 % CI: 0.84–1.21; *P* = 0.947; Table 2 and Additional file 2: Figure S1), with no evidence of heterogeneity among included studies . Furthermore, the pooled RRR (female to male) was 0.96 (95 % CI: 0.75–1.23; *P* = 0.772; Table 2 and Additional file 2: Figure S2) for moderate alcohol intake (15–30 g/day). There was a significant heterogeneity among the included studies (I^2^ = 40.7 %; *P* = 0.096). Finally, the pooled RRR (female to male) was reduced by 10 % (RRR, 0.90; 95 % CI: 0.66–1.22; *P* = 0.503; with moderate heterogeneity; Table 2 and Additional file 2: Figure S3) for heavy alcohol intake (>30 g/day), but this reduction was not statistically significant.

**Table 2:** Summary of the relative risks (ratios) of major cardiovascular outcomes and total mortality

Outcomes	Categories of alcohol intake	Men (RR and 95 % CI)	Women (RR and 95 % CI)	Women compared with men (RRR and 95 % CI)
Coronary disease	Low	0.67 (0.47–0.97)*	0.70 (0.51–0.97)*	1.01 (0.84–1.21)
	Moderate	0.68 (0.50–0.93)*	0.70 (0.52–0.94)*	0.96 (0.75–1.23)
	Heavy	0.69 (0.49–0.96)*	0.66 (0.44–0.99)*	0.90 (0.66–1.22)
Total mortality	Low	0.74 (0.60–0.92)*	0.87 (0.71–1.07)	1.07 (0.98–1.17)
	Moderate	0.80 (0.68–0.95)*	0.95 (0.83–1.08)	1.10 (1.00–1.21)*
	Heavy	1.00 (0.81–1.22)	1.20 (0.99–1.46)	1.09 (0.99–1.21)
Cardiac death	Low	0.93 (0.85–1.01)	0.84 (0.71–0.99)*	0.93 (0.83–1.04)
	Moderate	0.85 (0.78–0.92)*	0.86 (0.68–1.08)	0.99 (0.87–1.14)
	Heavy	0.93 (0.70–1.23)	1.04 (0.74–1.46)	1.14 (0.99–1.32)
Stroke	Low	0.89 (0.79–1.00)	0.89 (0.76–1.06)	0.99 (0.83–1.16)
	Moderate	0.91 (0.81–1.02)	0.79 (0.69–0.91)*	0.90 (0.74–1.10)
	Heavy	1.19 (0.93–1.52)	1.37 (0.92–2.04)	1.35 (0.77–2.35)
Ischemic stroke	Low	0.83 (0.69–0.99)*	0.79 (0.68–0.92)*	0.94 (0.74–1.20)
	Moderate	0.91 (0.77–1.08)	0.81 (0.67–0.96)*	0.88 (0.66–1.16)
	Heavy	1.18 (0.96–1.44)	1.12 (0.86–1.45)	1.04 (0.80–1.36)

*P < 0.05

### Total mortality

A total of 10 cohorts (14 studies) reported an association between alcohol intake and the risk of total mortality [8, 10, 25–29, 34–36, 40–43]. The summary RR of the associated between alcohol intake and total mortality in men and women, were separately listed in Table 2. The pooled RRR (female to male) for moderate alcohol intake and the risk of total mortality was statistically significantly increased (RRR, 1.10; 95 % CI: 1.00–1.21; *P* = 0.047; Table 2 and Additional file 2: Figure S5). Although the summary RRR (female to male) increased, there was no significant association between low (RRR, 1.07; 95 % CI: 0.98–1.17; *P* = 0.143; Table 2 and Additional file 2: Figure S4) or heavy alcohol intake (RRR, 1.09; 95 % CI: 0.99–1.21; *P* = 0.084; Table 2 and Additional file 2: Figure S6) and the risk of total mortality in women compared with men.

### Cardiac death, stroke, and ischemic stroke

The breakdown for the number of cohorts available for each outcome were four (seven studies), five (seven studies), and three (five studies) for cardiac death [25, 31, 34–36, 42, 43], stroke [10, 30, 34–38], and ischemic stroke [24, 30, 34–36] respectively. These associations in men and women separately were shown in Table 2. The summary RRRs (female to male) of low alcohol intake were 0.93, 0.99, and 0.94 for cardiac death (RRR, 0.93; 95 % CI: 0.83–1.04; *P* = 0.216; Table 2 and Additional file 2: Figure S8), stroke (RRR, 0.99; 95 % CI: 0.83–1.16; *P* = 0.864; Table 2 and Additional file 2: Figure S9), and ischemic stroke (RRR, 0.94; 95 % CI: 0.74–1.20; *P* = 0.633; Table 2 and Additional file 2: Figure S7) respectively. Similarly, the summary RRRs (female to male) of moderate alcohol intake were 0.99, 0.90, and 0.88 for cardiac death (RRR, 0.99; 95 % CI: 0.87–1.14; *P* = 0.934; Table 2 and Additional file 2: Figure S10), stroke (RRR, 0.90; 95 % CI: 0.74–1.10; *P* = 0.299; Table 2 and Additional file 2: Figure S11), and ischemic stroke (RRR, 0.88; 95 % CI: 0.66–1.16; *P* = 0.366; Table 2 and Additional file 2: Figure S12) respectively. Finally, the summary RRRs (female to male) of low alcohol intake were 1.44, 1.35, and 1.04 for cardiac death (RRR, 1.14; 95 % CI: 0.99–1.32; *P* = 0.075; Table 2 and Additional file 2: Figure S13), stroke (RRR, 1.35; 95 % CI: 0.77–2.35; *P* = 0.292; Table 2 and Additional file 2: Figure S14), and ischemic stroke (RRR,1.04; 95 % CI: 0.80–1.36; *P* = 0.762; Table 2 and Additional file 2: Figure S15) respectively.

### Sensitivity analysis and subgroup analysis

Sensitivity analyses indicated that exclusion of any individual study did not significantly alter the results (data not shown). Heterogeneity testing for the analysis showed P >0.10 for coronary disease and total mortality. We concluded that heterogeneity was not significant in the overall analysis, which suggested that most variation was attributable to chance alone. Subgroup analyses were also conducted for coronary disease and total mortality to evaluate the effect of alcohol intake in women compared with men in specific subpopulations. The summary RRR (female to male) was significantly increased for the association between moderate alcohol intake and the risk of coronary disease for studies conducted in the US. Furthermore, the summary RRR (female to male) was significantly increased for the association between heavy alcohol intake and subsequent total mortality risk for studies conducted in the US. The study was not adjusted for physical activity, diabetes, or low NOS score (Table 3).

**Table 3:** Subgroup analysis of coronary disease and total mortality of alcohol intake versus the lowest intake for women compared with men

Outcomes	Subgroup	Low alcohol intake	Moderate alcohol intake	Heavy alcohol intake
Coronary disease	Country			
	US	1.24 (0.72–2.13)	2.17 (1.12–4.21)*	1.25 (0.52–3.01)
	Other	0.98 (0.80–1.20)	0.89 (0.75–1.23)	0.87 (0.62–1.22)
	Sample size			
	>10000	0.96 (0.80–1.16)	0.98 (0.73–1.30)	0.89 (0.63–1.25)
	<10000	1.42 (0.82–2.45)	0.93 (0.49–1.78)	1.00 (0.40–2.49)
	Adjusted physical activity			
	Yes	1.01 (0.80–1.27)	0.86 (0.66–1.12)	0.85 (0.65–1.11)
	No	0.96 (0.63–1.48)	1.24 (0.75–2.05)	0.91 (0.35–2.38)
	Adjusted serum cholesterol			
	Yes	1.08 (0.84–1.38)	0.96 (0.72–1.28)	1.20 (0.80–1.78)
	No	0.93 (0.68–1.27)	1.00 (0.64–1.57)	0.75 (0.52–1.08)
	Adjusted hypertension			
	Yes	1.09 (0.85–1.40)	0.76 (0.49–1.17)	1.10 (0.68–1.80)
	No	0.92 (0.68–1.26)	1.11 (0.83–1.48)	0.77 (0.51–1.15)
	Adjusted diabetes			
	Yes	0.99 (0.78–1.27)	0.82 (0.61–1.09)	0.86 (0.66–1.12)
	No	1.01 (0.72–1.40)	1.22 (0.84–1.78)	0.83 (0.35–1.97)
	Follow-up duration			
	More than 10 years	1.01 (0.72–1.40)	1.09 (0.83–1.43)	0.86 (0.49–1.54)
	Less than 10 years	0.99 (0.78–1.27)	0.75 (0.48–1.17)	0.87 (0.63–1.21)
	Study quality (NOS score)			
	8 or 9	0.76 (0.50–1.16)	0.93 (0.74–1.17)	0.70 (0.42–1.17)
	<8	1.12 (0.90–1.39)	0.98 (0.63–1.54)	1.09 (0.76–1.55)
Total mortality	Country			
	US	1.06 (0.94–1.20)	1.14 (0.97–1.33)	1.16 (1.02–1.33)*
	Other	1.08 (0.94–1.24)	1.07 (0.96–1.20)	1.01 (0.87–1.18)
	Sample size			
	>10000	1.06 (0.96–1.16)	1.09 (0.99–1.19)	1.08 (0.98–1.20)
	<10000	1.36 (0.91–2.01)	1.25 (0.77–2.03)	1.63 (0.87–3.06)
	Adjusted physical activity			
	Yes	1.10 (0.96–1.26)	1.08 (0.96–1.20)	1.02 (0.87–1.18)
	No	1.05 (0.93–1.18)	1.13 (0.97–1.32)	1.16 (1.01–1.33)*
	Adjusted serum cholesterol			
	Yes	1.15 (0.73–1.80)	1.21 (0.79–1.86)	1.28 (0.61–2.65)
	No	1.07 (0.97–1.17)	1.09 (0.99–1.19)	1.09 (0.98–1.21)
	Adjusted hypertension			
	Yes	1.08 (0.94–1.24)	1.08 (0.87–1.34)	1.07 (0.83–1.37)
	No	1.06 (0.94–1.20)	1.10 (0.99–1.21)	1.10 (0.98–1.23)
	Adjusted diabetes			
	Yes	1.08 (0.93–1.24)	1.07 (0.96–1.20)	1.01 (0.87–1.18)
	No	1.07 (0.95–1.20)	1.13 (0.97–1.31)	1.16 (1.02–1.33)*
	Follow-up duration			
	More than 10 years	1.08 (0.98–1.19)	1.09 (0.99–1.20)	1.10 (0.99–1.22)
	Less than 10 years	0.99 (0.72–1.37)	1.24 (0.76–2.03)	1.04 (0.76–1.43)
	Study quality (NOS score)			
	8 or 9	1.08 (0.95–1.23)	1.08 (0.97–1.21)	1.03 (0.88–1.20)
	<8	1.06 (0.93–1.21)	1.11 (0.95–1.30)	1.15 (1.00–1.31)*

### Publication bias

Review of the funnel plots could not rule out the potential for publication bias for the risk of coronary disease, and total mortality. The Egger [22] and Begg test [23] results showed no evidence of publication bias for the risk of coronary disease (low [Additional file 2: Figure S16], moderate [Additional file 2: Figure S17] and heavy alcohol intake [Additional file 2: Figure S18]), total mortality (low [Additional file 2: Figure S16], moderate [Additional file 2: Figure S17] and heavy alcohol intake [Additional file 2: Figure S18]) in women compared with men.

## Discussion

Our current study was based on prospective observational studies and was used to explore all possible correlations between alcohol intake and coronary disease, total mortality, cardiac death, stroke, or ischemic stroke in women compared with men. This large quantitative study included 489,696 individuals from 18 prospective cohorts across a broad range of populations. Under the condition of without considering other independent cardiovascular risk factors, the findings of this meta-analysis indicated that female with moderate alcohol intake had a 10 % greater RR of total mortality than male drinkers. Furthermore, subgroup analyses indicated that among US participants, women with moderate alcohol intake had an increased risk of coronary disease (117 %) compared to male drinkers, and that women with heavy alcohol intake had a 16 %, 16 %, 16 %, and 15 % greater RR of total mortality than male drinkers for the study conducted in US, the study not adjusted for physical activity, the study not adjusted for diabetes, or the study with low NOS score respectively.

A previous meta-analysis [44] suggested that the increased alcohol intake was associated with a reduced risk of coronary disease in men and women, but there was no significant difference in the effect of alcohol intake and subsequent coronary disease risk between men and women. The inherent limitation of that previous review was that the study did not provide the results of gender difference. The current study indicated that low-to-heavy alcohol intake might be protective against coronary disease risk in men and women, separately. Furthermore, although the summary RRR (female to male) was slight reduced for the association between moderate or heavy alcohol intake and the risk of coronary disease in women compared with men, the reduction was not statistically significant. Finally, subgroup analyses indicated that there was no statistical evidence for differing beneficial effects of alcohol intake and subsequent risk of coronary disease between men and women, except in US participants. A possible reason for this could be that women had a lower gastric alcohol dehydrogenase activity, resulting in higher blood ethanol levels [45]. Furthermore, while the subgroup analysis indicated that US women with moderate alcohol intake had a 117 % greater RR, it might be unreliable because that the analysis only included one study.

In a meta-analysis, Castelnuovo et al. [46] indicated that low alcohol intake was associated with reduced risk of total mortality in both men and women, while heavy alcohol intake was associated with increased risk of total mortality. Costanzo et al. [47] suggested that low-to-moderate alcohol intake was significantly associated with a lower incidence of total mortality. The current study suggested that low-to-moderate alcohol might protect against total mortality risk in men, whereas there was no significant effect on the risk of total mortality in women. Furthermore, women with moderate alcohol intake had a 10 % greater RR for total mortality than men. The possible reasons for this were as follows: (1) there were multiple interrelations between alcohol intake and other risk factors of total mortality. In the current study, subgroup analysis showed that these associations differed if adjustments were made for physical activity or diabetes. However, we could not determine the effects of these potential confounding factors on the risk of total mortality because that very few studies were stratified using these confounders; (2) women with alcohol intake had higher blood ethanol levels, resulting in higher risk of liver disease, which significantly increases the risk of total mortality [45]; (3) concerns remained regarding the impact of the association between the pattern and duration of alcohol intake, such as binge drinking, and the risk of total mortality. Unfortunately, data on the pattern and duration of alcohol intake were rarely available in these studies, therefore, no conclusions could be made.

Previous meta-analyses suggested that increased alcohol intake was associated with a 23 % and 22 % reduction in risk of cardiac death for men and women, respectively [44]. Similarly, in the current study, no significant differences were observed in the relative risk ratios of cardiac death between those men and women who consumed alcohol. Women included in our study were mostly postmenopausal and had a higher incidence of cardiovascular disease; additionally, the association between alcohol and risk of cardiac death might appear to be reduced by regular drinking habits in women [48]. Furthermore, only three prospective observational studies reported the effect estimates of cardiac death for men and women separately. This conclusion might have not been accurate since smaller cohorts were included.

A previous meta-analysis conducted by Zhang et al. [49] illustrated the association between alcohol intake and the risk of stroke and stroke somatotypes, and suggested that low alcohol intake was associated with a reduced risk of stroke morbidity and mortality, whereas heavy alcohol intake was associated with an increased risk of total stroke. Furthermore, another important meta-analysis indicated that alcohol intake was associated with stroke risk; the study reported a 2 % risk increase and a 13 % risk reduction for men and women, respectively. In this study, there was no significant difference between men and women for the association between alcohol intake and the risk of stroke or ischemic stroke. Although women who consumed alcohol had a higher RR of stroke or ischemic stroke than men, this difference might be due to chance, as fewer studies were included, which might have resulted in less variation in the conclusions.

Two strengths of our study should be highlighted. First, only prospective studies were included, which should eliminate selection and recall bias that might be concerned of retrospective case–control studies. Second, the large sample size allowed us to quantitatively assess the gender difference for the association between alcohol intake and the risk of major cardiovascular outcomes or total mortality, and thus, our findings were potentially more robust than those of any individual study.

The limitations of our study were as follows: (1) the cut-off points for the alcohol intake categories differed among studies; (2) in a meta-analysis of published studies, publication bias was an inevitable problem; (3) the validity of self-reported alcohol intake during the follow-up period could be questioned; and (4) the analysis used pooled data (individual data were not available), which restricted us from performing a more detailed relevant analysis and obtaining more comprehensive results.

## Conclusions

The results of this study indicated that women who were conferred by moderate alcohol intake had a significant 10 % increased risk of total mortality compared with men. Furthermore, stratified analyses suggested that women with heavy alcohol intake had a significantly increased RR risk of total mortality than male drinkers in multiple subpopulations. Future studies should focus on specific populations, especially for patients with chronic diseases in order to evaluate the secondary prevention of major cardiovascular outcomes.

## Additional files

Additional file 1:
**Search strategy.** (DOCX 13 kb) Additional file 2: Table S1. Adjustment factors of included studies. **Figures S1.** RR or RRR (female to male) of low alcohol intake and the risk of coronary disease. **Figure S2.** RR or RRR (female to male) of moderate alcohol intake and the risk of coronary disease. **Figure S3.** RR or RRR (female to male) of heavy alcohol intake and the risk of coronary disease. **Figure S4.** RR or RRR (female to male) of low alcohol intake and the risk of total mortality. **Figure S5.** RR or RRR (female to male) of moderate alcohol intake and the risk of total mortality. **Figure S6.** RR or RRR (female to male) of heavy alcohol intake and the risk of total mortality. **Figure S7.** RR or RRR (female to male) of low alcohol intake and the risk of ischemic stroke. **Figure S8.** RR or RRR (female to male) of low alcohol intake and the risk of cardiac death. **Figure S9.** RR or RRR (female to male) of low alcohol intake and the risk of stroke. **Figure S10.** RR or RRR (female to male) of moderate alcohol intake and the risk of cardiac death. **Figure S11.** RR or RRR (female to male) of moderate alcohol intake and the risk of stroke. **Figure S12.** RR or RRR (female to male) of moderate alcohol intake and the risk of ischemic stroke. **Figure S13.** RR or RRR (female to male) of heavy alcohol intake and the risk of cardiac death. **Figure S14.** RR or RRR (female to male) of heavy alcohol intake and the risk of stroke. **Figure S15.** RR or RRR (female to male) of heavy alcohol intake and the risk of ischemic stroke. **Figure S16.** Funnel plot of RRR (female to male) for low alcohol intake. **Figure S17.** Funnel plot of RRR (female to male) for moderate alcohol intake. **Figure S18.** Funnel plot of RRR (female to male) for heavy alcohol intake. (DOC 10344 kb)
