# Supplementary material for: Alcohol intake and associated risk of major cardiovascular outcomes in women compared with men: a systematic review and meta-analysis of prospective observational studies
Source: BMC Public Health. 2015 Aug 12;15:773. doi: 10.1186/s12889-015-2081-y (PMC4533962; doi:10.1186/s12889-015-2081-y)
Supplement: Additional file 1: — Search strategy. (DOCX 13 kb) [file 12889_2015_2081_MOESM1_ESM.docx]

**Search terms:**

1. ethanol

2. alcohol

3. alcoholic beverages

4. drinking behavior

5. alcohol drinking

6. #1 OR #2 OR #3 OR #4 OR #5

7. stroke

8. cardiovascular diseases

9. myocardial infarction

10. myocardial ischemia

11. coronary artery disease

12. heart infarction

13. #7 OR #8 OR #9 OR #10 OR #11 OR #12

14. #6 AND #13

15. #14 AND 'human' AND ('nested case control study'/de OR 'clinical trial'/de OR 'cohort analysis'/de OR 'prospective study'/de)
