# Supplementary material for: Alcohol intake and associated risk of major cardiovascular outcomes in women compared with men: a systematic review and meta-analysis of prospective observational studies
Source: BMC Public Health. 2015 Aug 12;15:773. doi: 10.1186/s12889-015-2081-y (PMC4533962; doi:10.1186/s12889-015-2081-y)
Supplement: Additional file 2: Table S1. — Adjustment factors of included studies. Figures S1. RR or RRR (female to male) of low alcohol intake and the risk of coronary disease. Figure S2. RR or RRR (female to male) of moderate alcohol intake and the risk of coronary disease. Figure S3. RR or RRR (female to male) of heavy alcohol intake and the risk of coronary disease. Figure S4. RR or RRR (female to male) of low alcohol intake and the risk of total mortality. Figure S5. RR or RRR (female to male) of moderate alcohol intake and the risk of total mortality. Figure S6. RR or RRR (female to male) of heavy alcohol intake and the risk of total mortality. Figure S7. RR or RRR (female to male) of low alcohol intake and the risk of ischemic stroke. Figure S8. RR or RRR (female to male) of low alcohol intake and the risk of cardiac death. Figure S9. RR or RRR (female to male) of low alcohol intake and the risk of stroke. Figure S10. RR or RRR (female to male) of moderate alcohol intake and the risk of cardiac death. Figure S11. RR or RRR (female to male) of moderate alcohol intake and the risk of stroke. Figure S12. RR or RRR (female to male) of moderate alcohol intake and the risk of ischemic stroke. Figure S13. RR or RRR (female to male) of heavy alcohol intake and the risk of cardiac death. Figure S14. RR or RRR (female to male) of heavy alcohol intake and the risk of stroke. Figure S15. RR or RRR (female to male) of heavy alcohol intake and the risk of ischemic stroke. Figure S16. Funnel plot of RRR (female to male) for low alcohol intake. Figure S17. Funnel plot of RRR (female to male) for moderate alcohol intake. Figure S18. Funnel plot of RRR (female to male) for heavy alcohol intake. (DOC 10344 kb) [file 12889_2015_2081_MOESM2_ESM.doc]

Table S1. adjustment factors of included studies.

| Study | Age | BMI | Serum cholesterol | Smoking | Diabetes | Physical activity | Hypertension |
| --- | --- | --- | --- | --- | --- | --- | --- |
| Framingham | Yes | Yes | No | Yes | Yes | No | No |
| DANCOS | No | Yes | No | Yes | Yes | Yes | Yes |
| EPOZ | Yes | Yes | Yes | Yes | No | No | Yes |
| MPC | Yes | Yes | No | Yes | No | No | No |
| NHEFS | Yes | Yes | No | Yes | No | Yes | No |
| Whitehall II | Yes | Yes | Yes | Yes | No | No | Yes |
| MONICA/KORA-Augsburg | Yes | Yes | Yes | Yes | No | Yes | Yes |
| MCCS | Yes | No | No | Yes | No | No | No |
| CCHS | Yes | Yes | No | Yes | Yes | Yes | No |
| HPFS and NHS | Yes | No | No | No | No | No | No |
| LWCS | Yes | Yes | No | Yes | Yes | Yes | Yes |
| DDCHS | Yes | Yes | Yes | Yes | Yes | Yes | No |
| JACC | Yes | Yes | No | Yes | Yes | Yes | Yes |
| EPIC-Nutrition-Heidelberg | No | Yes | No | Yes | No | Yes | No |
| EPIC-Potsdam | Yes | Yes | Yes | Yes | Yes | Yes | Yes |
| EPIC-Spanish | Yes | No | No | Yes | No | Yes | No |
| Lifestyle and Health Study | Yes | Yes | Yes | Yes | Yes | Yes | Yes |
| NHIS | No | Yes | No | Yes | No | No | No |

**Supporting Figure Legends:**

Figure S1. RR or RRR (female to male) of low alcohol intake and the risk of coronary disease.

Figure S2. RR or RRR (female to male) of moderate alcohol intake and the risk of coronary disease.

Figure S3. RR or RRR (female to male) of heavy alcohol intake and the risk of coronary disease.

Figure S4. RR or RRR (female to male) of low alcohol intake and the risk of total mortality.

Figure S5. RR or RRR (female to male) of moderate alcohol intake and the risk of total mortality.

Figure S6. RR or RRR (female to male) of heavy alcohol intake and the risk of total mortality.

Figure S7. RR or RRR (female to male) of low alcohol intake and the risk of ischemic stroke.

Figure S8. RR or RRR (female to male) of low alcohol intake and the risk of cardiac death.

Figure S9. RR or RRR (female to male) of low alcohol intake and the risk of stroke.

Figure S10. RR or RRR (female to male) of moderate alcohol intake and the risk of cardiac death.

Figure S11. RR or RRR (female to male) of moderate alcohol intake and the risk of stroke.

Figure S12. RR or RRR (female to male) of moderate alcohol intake and the risk of ischemic stroke.

Figure S13. RR or RRR (female to male) of heavy alcohol intake and the risk of cardiac death.

Figure S14. RR or RRR (female to male) of heavy alcohol intake and the risk of stroke.

Figure S15. RR or RRR (female to male) of heavy alcohol intake and the risk of ischemic stroke.

Figure S16. Funnel plot of RRR (female to male) for low alcohol intake.

Figure S17. Funnel plot of RRR (female to male) for moderate alcohol intake.

Figure S18. Funnel plot of RRR (female to male) for heavy alcohol intake.


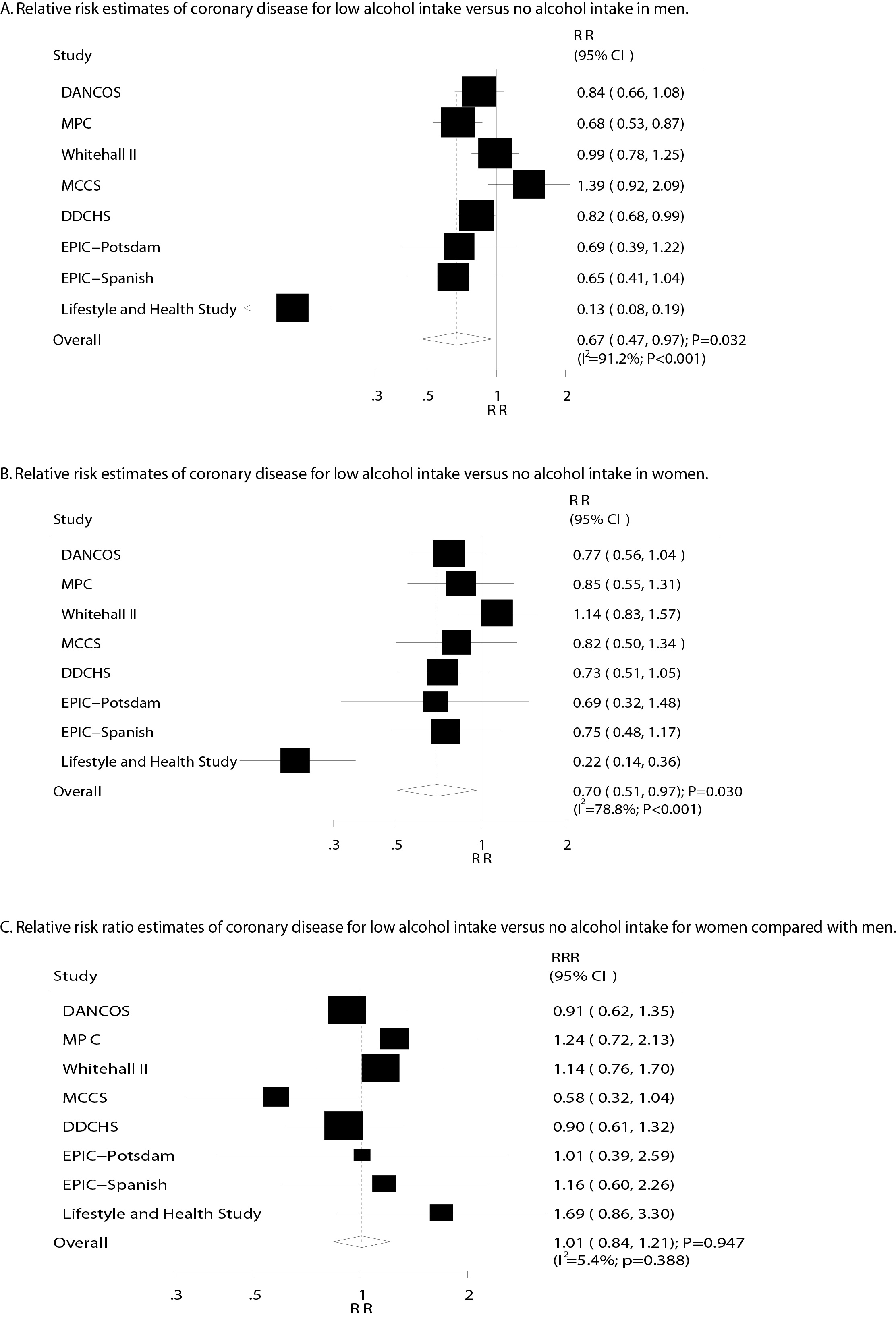


Figure S1. RR or RRR (female to male) of low alcohol intake and the risk of coronary disease.


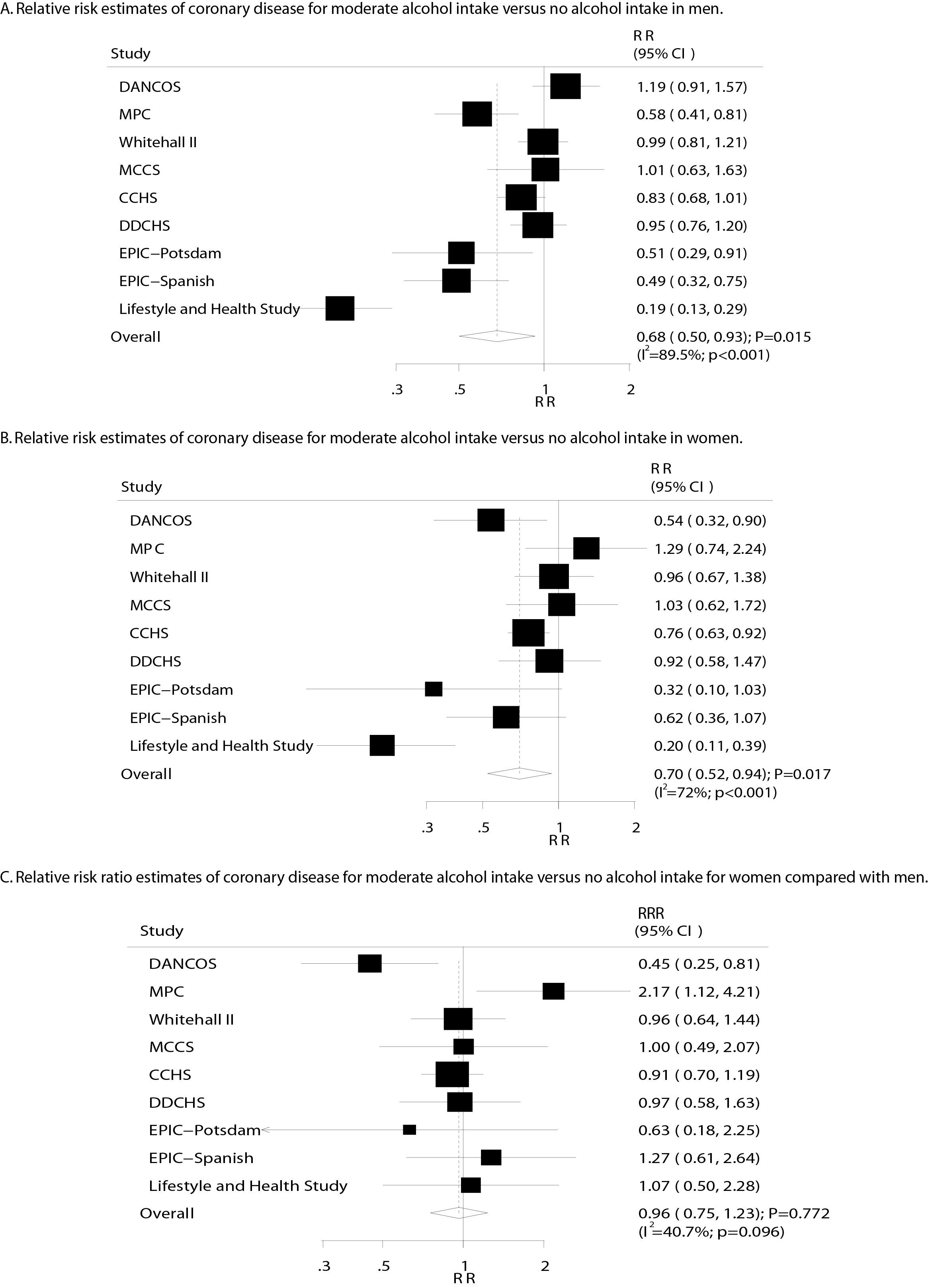


Figure S2. RR or RRR (female to male) of moderate alcohol intake and the risk of coronary disease.


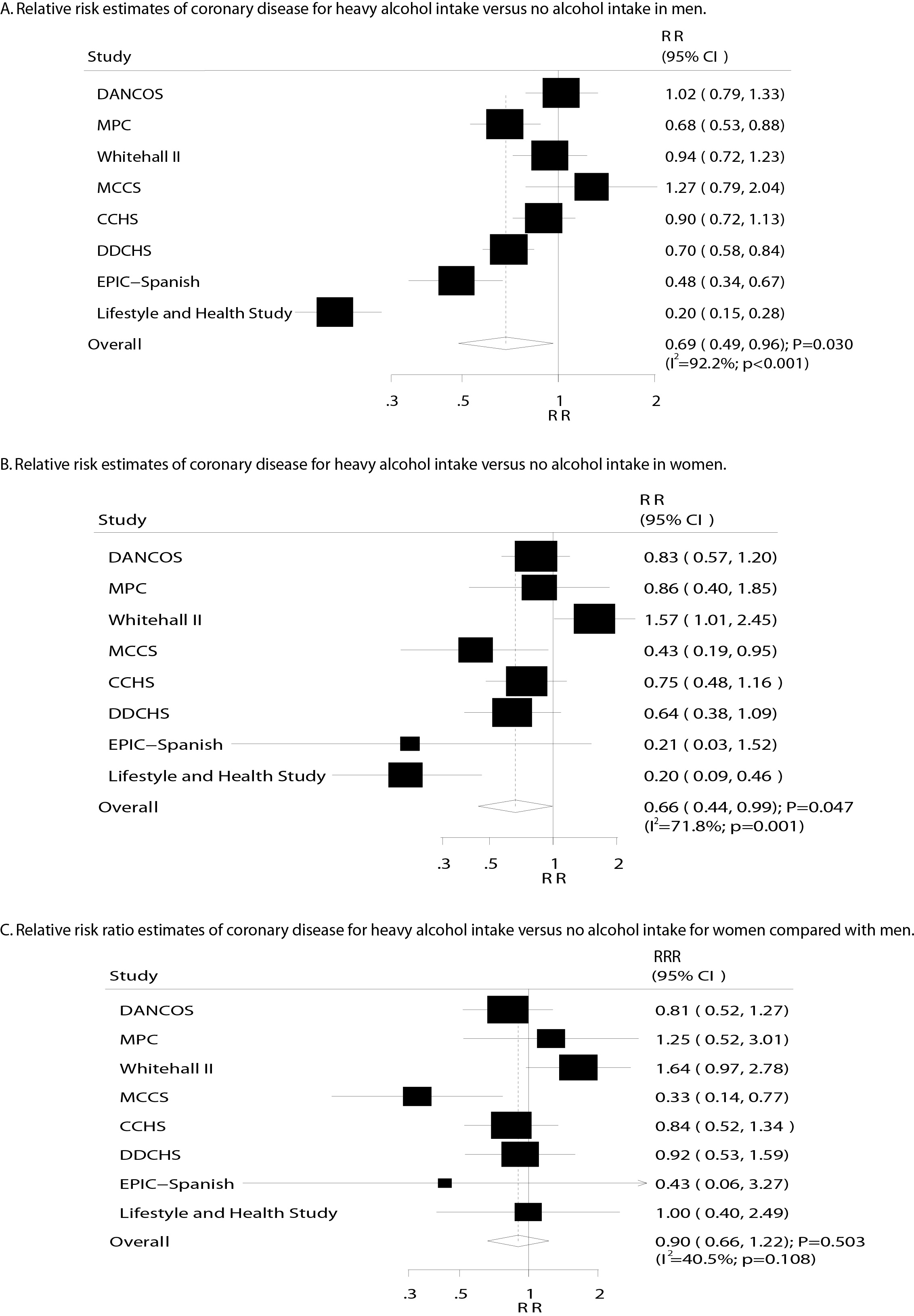


Figure S3. RR or RRR (female to male) of heavy alcohol intake and the risk of coronary disease.


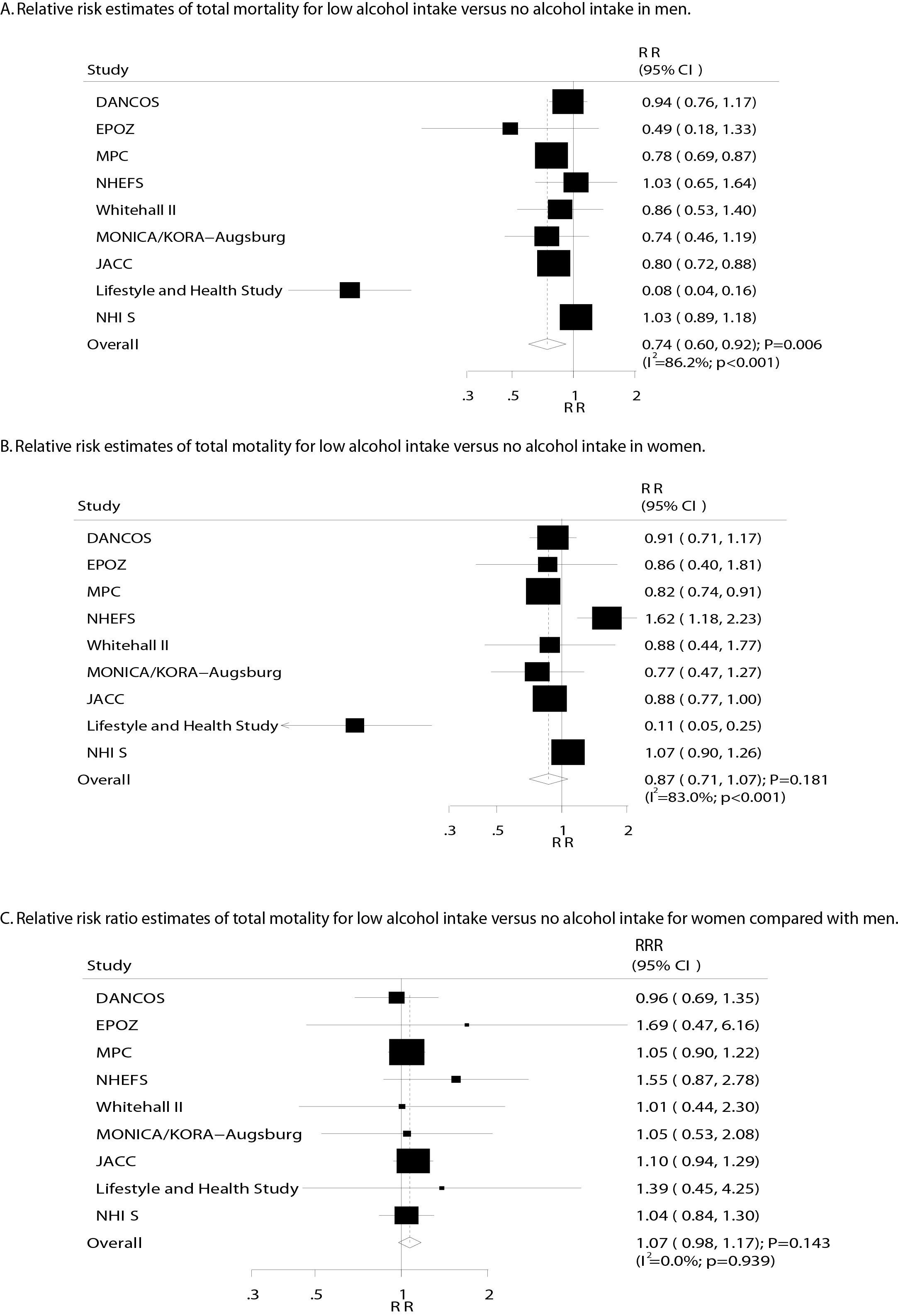


Figure S4. RR or RRR (female to male) of low alcohol intake and the risk of total mortality.


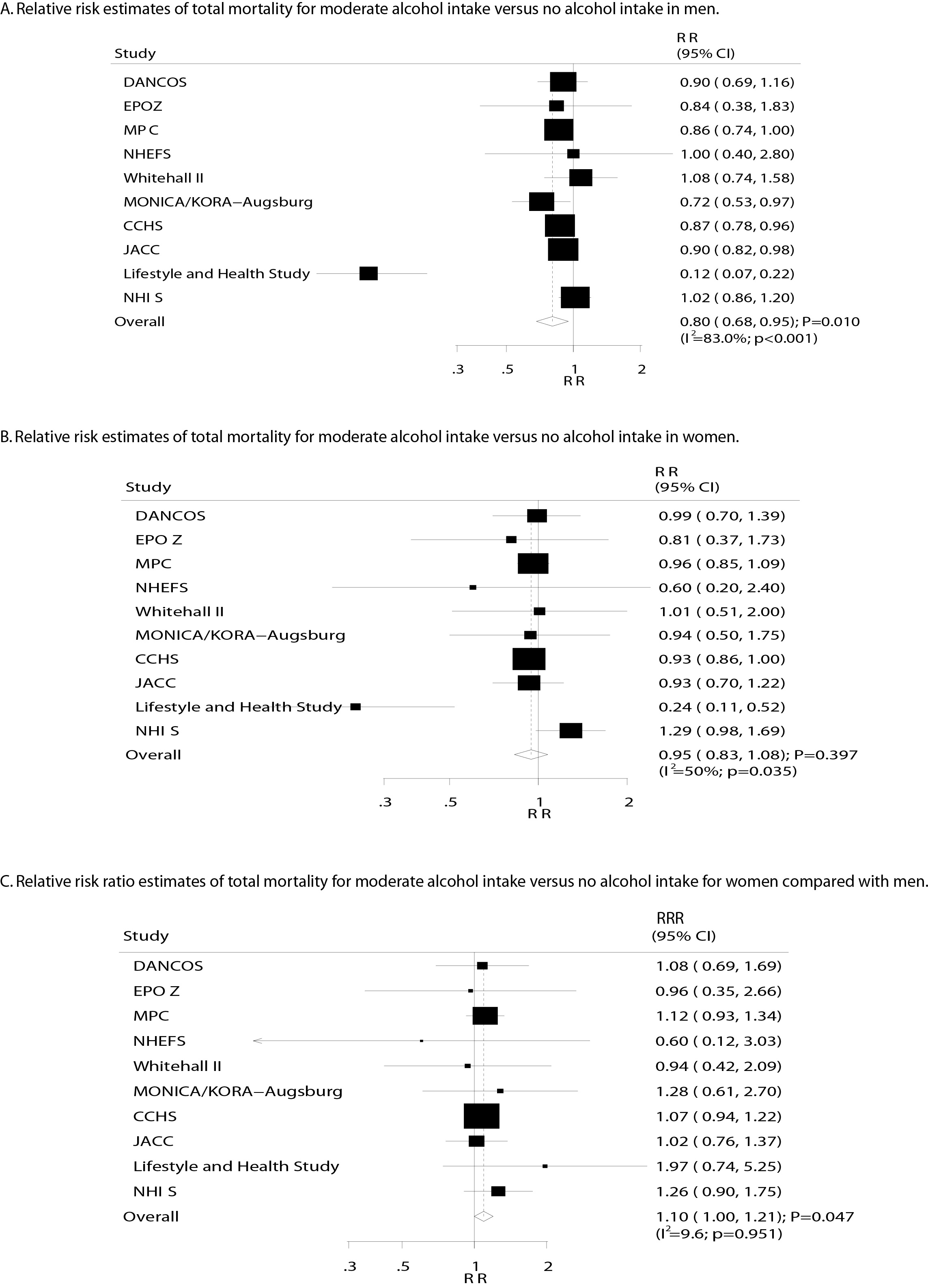


Figure S5. RR or RRR (female to male) of moderate alcohol intake and the risk of total mortality.


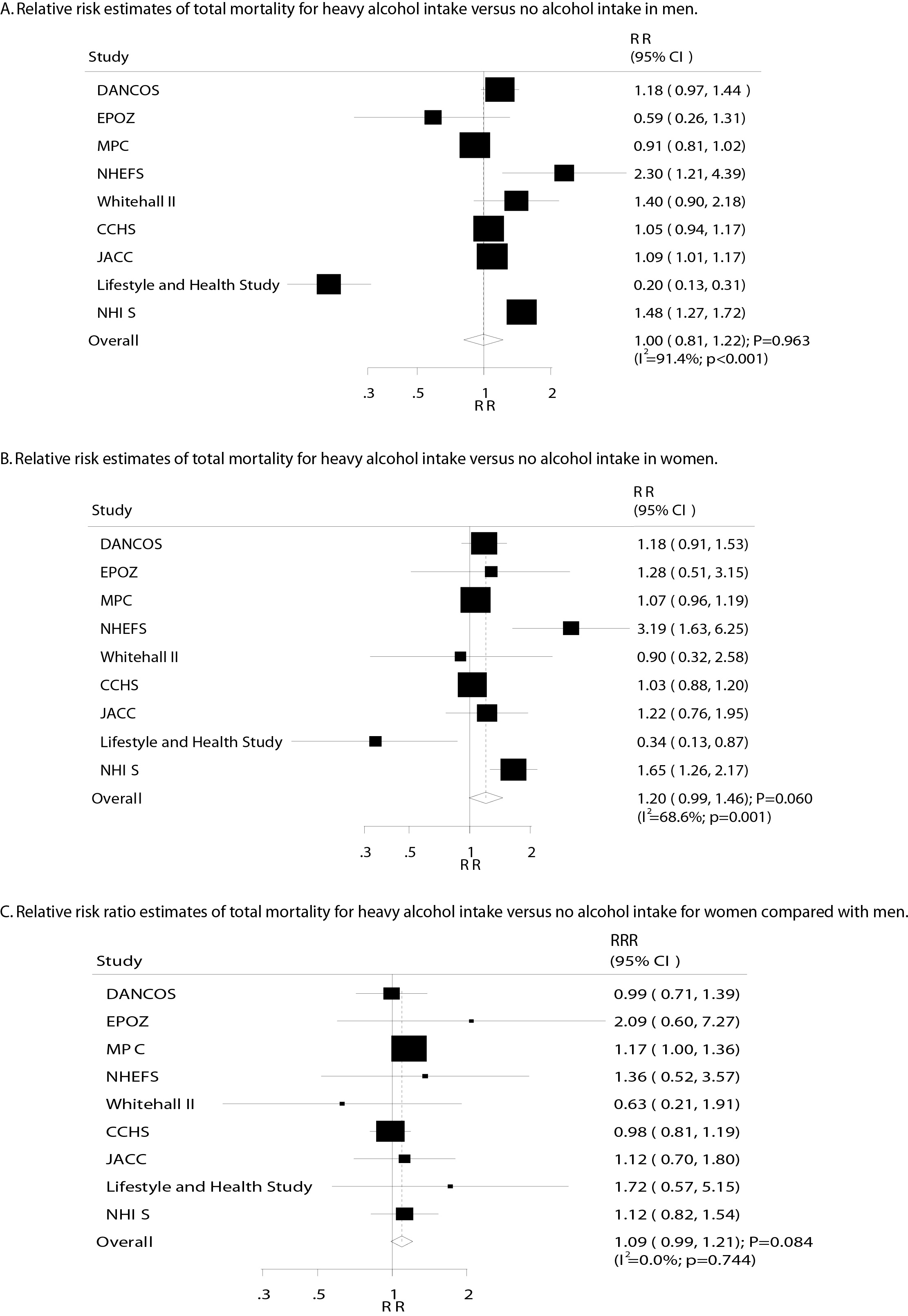


Figure S6. RR or RRR (female to male) of heavy alcohol intake and the risk of total mortality.


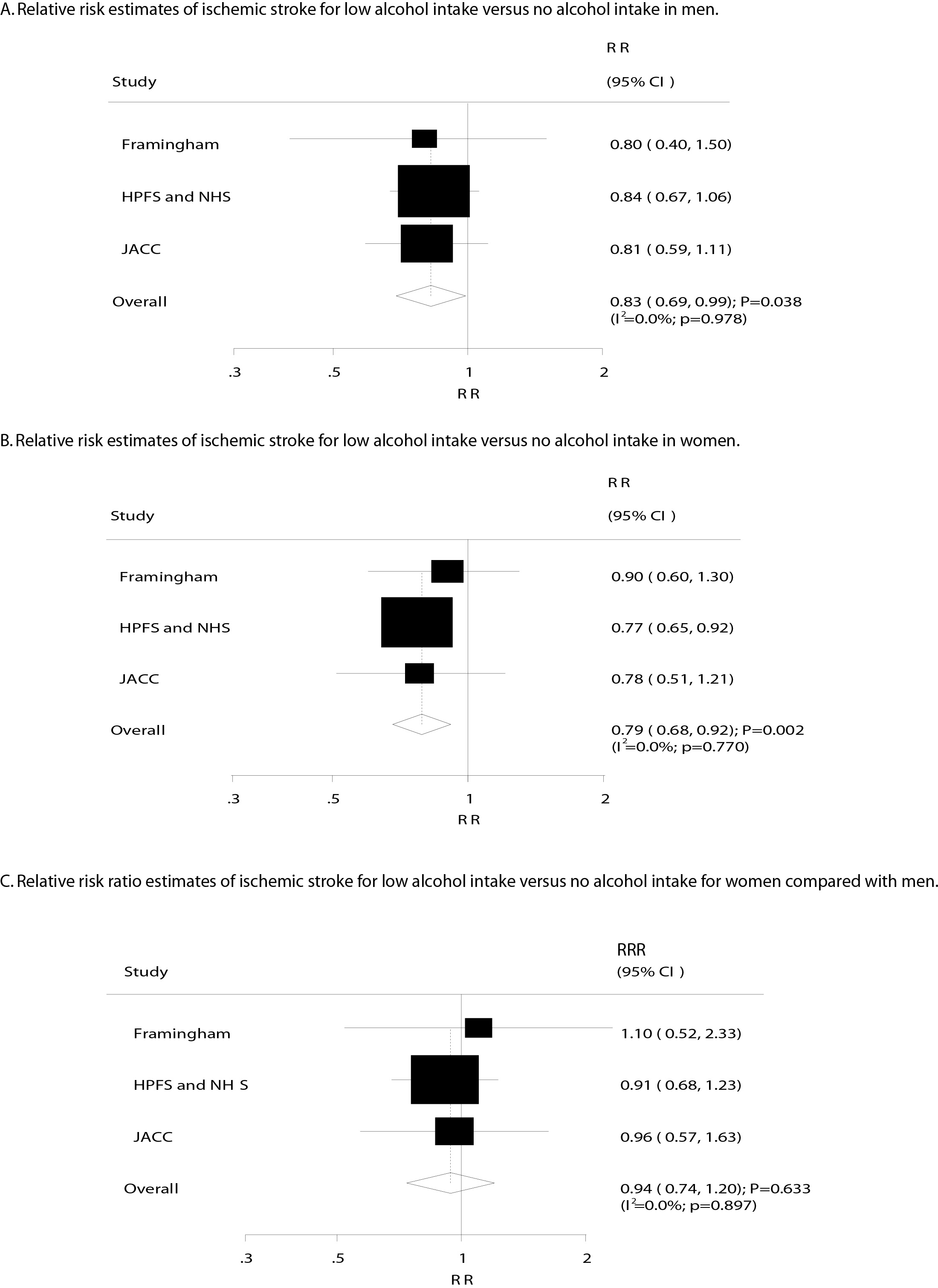


Figure S7. RR or RRR (female to male) of low alcohol intake and the risk of ischemic stroke.


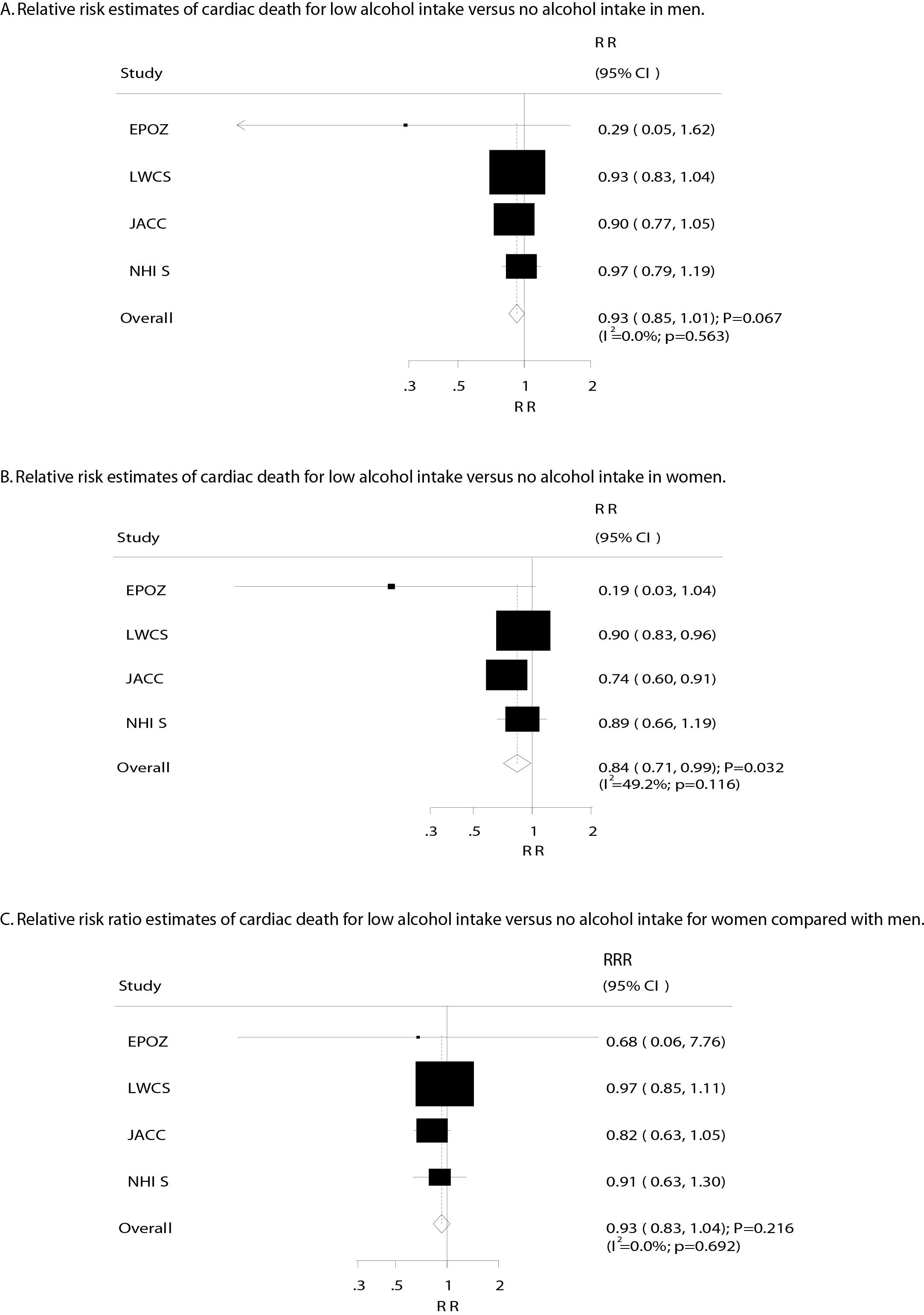


Figure S8. RR or RRR (female to male) of low alcohol intake and the risk of cardiac death.


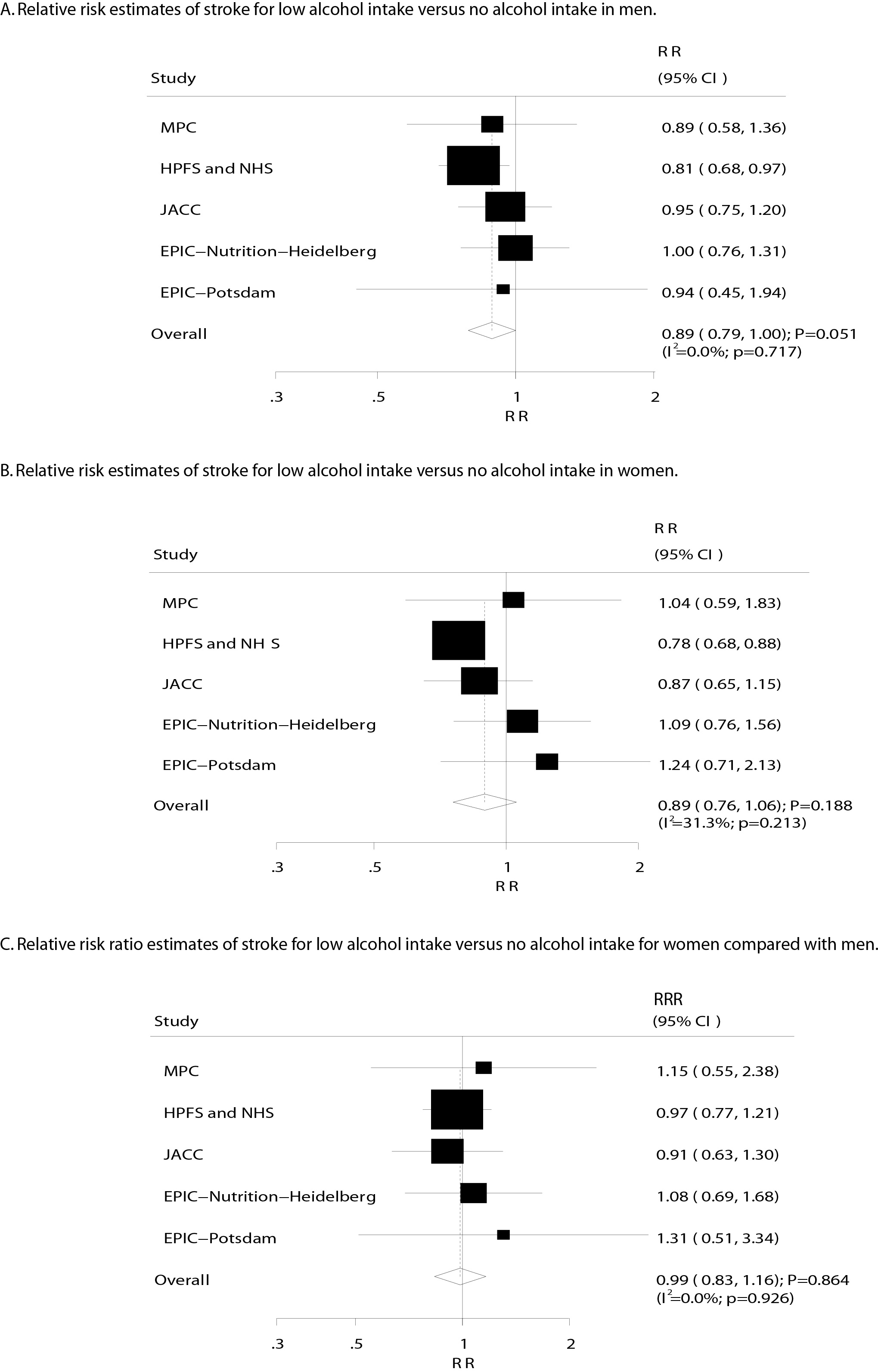


Figure S9. RR or RRR (female to male) of low alcohol intake and the risk of stroke.


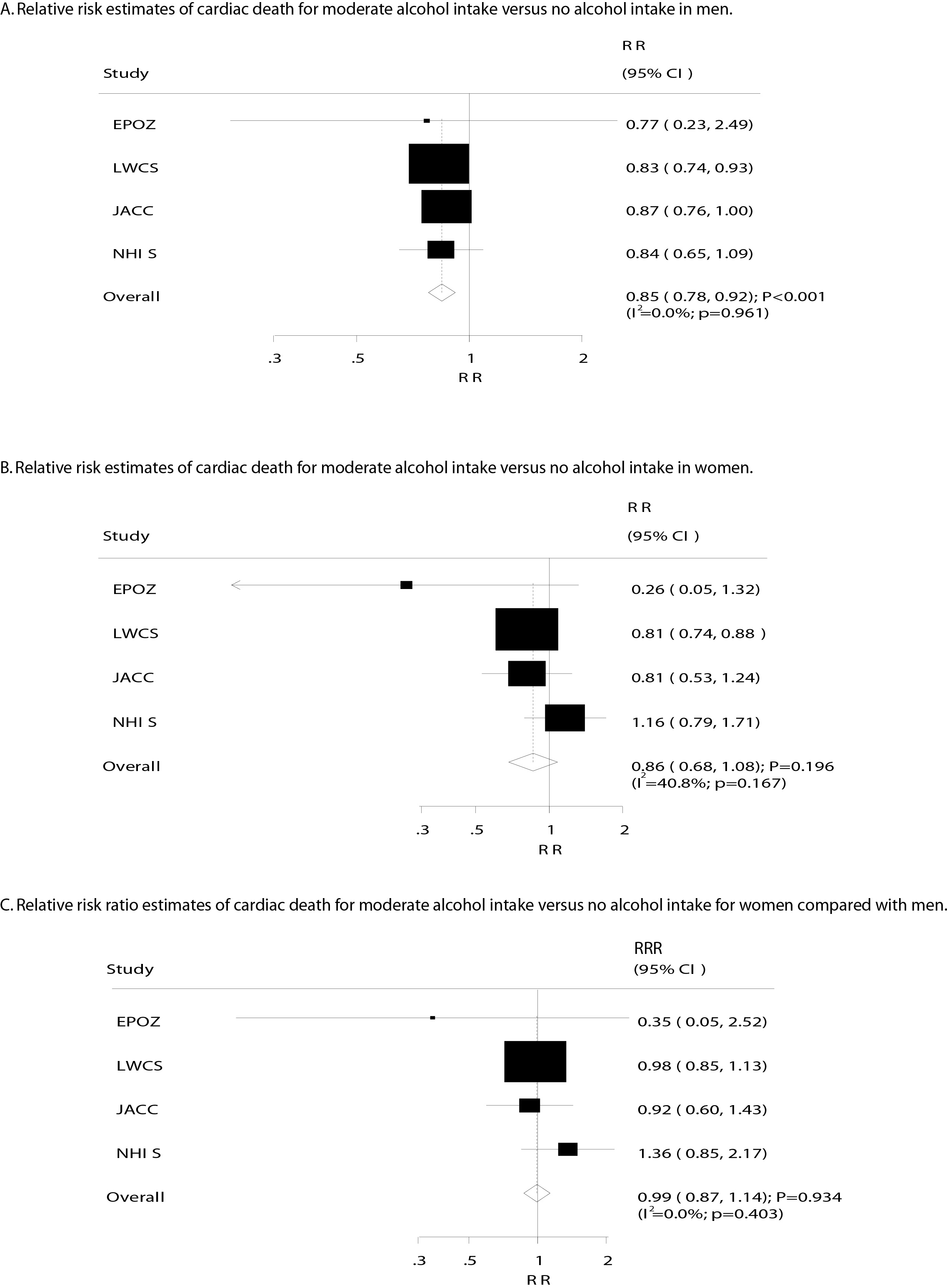


Figure S10. RR or RRR (female to male) of moderate alcohol intake and the risk of cardiac death.


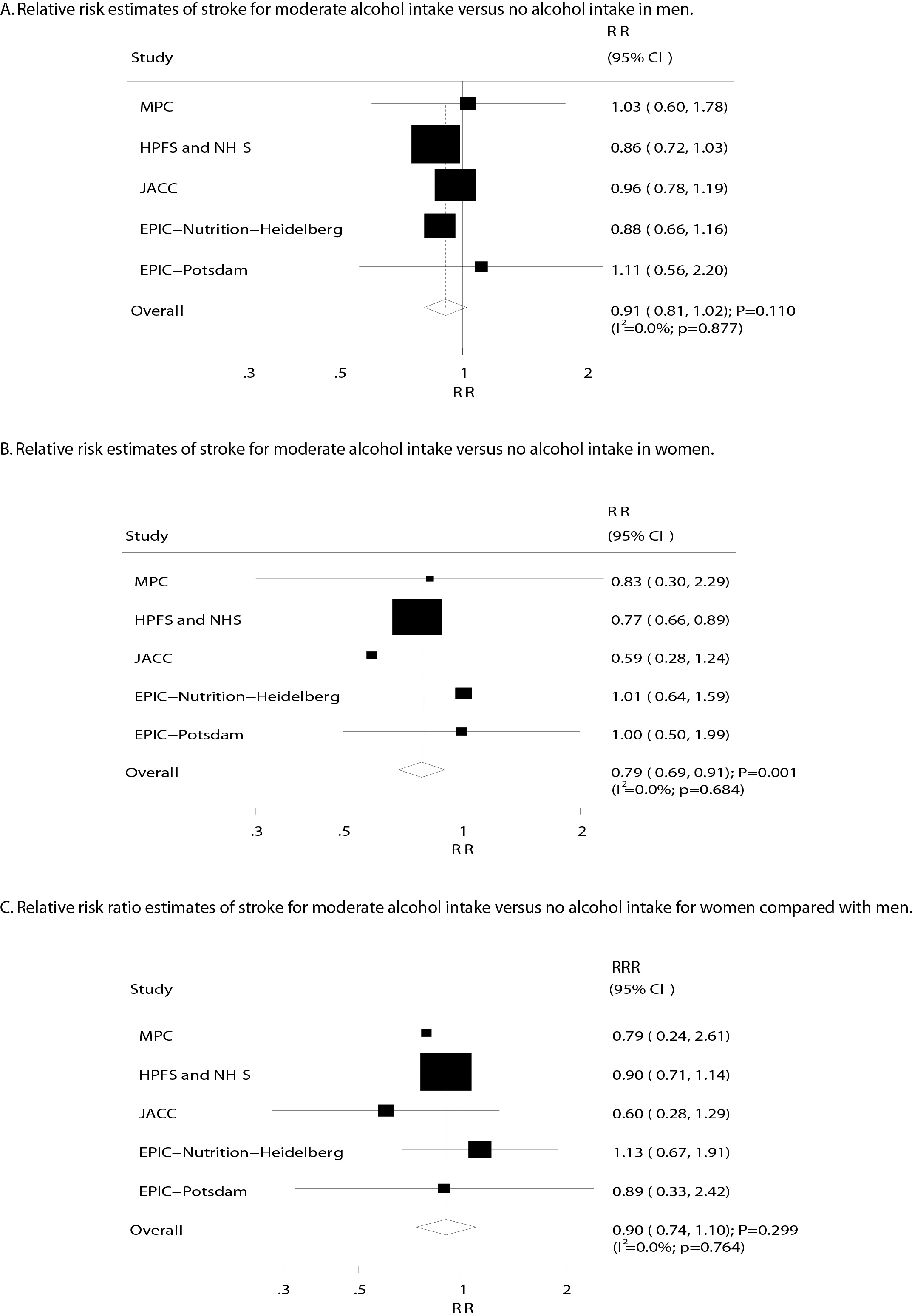


Figure S11. RR or RRR (female to male) of moderate alcohol intake and the risk of stroke.


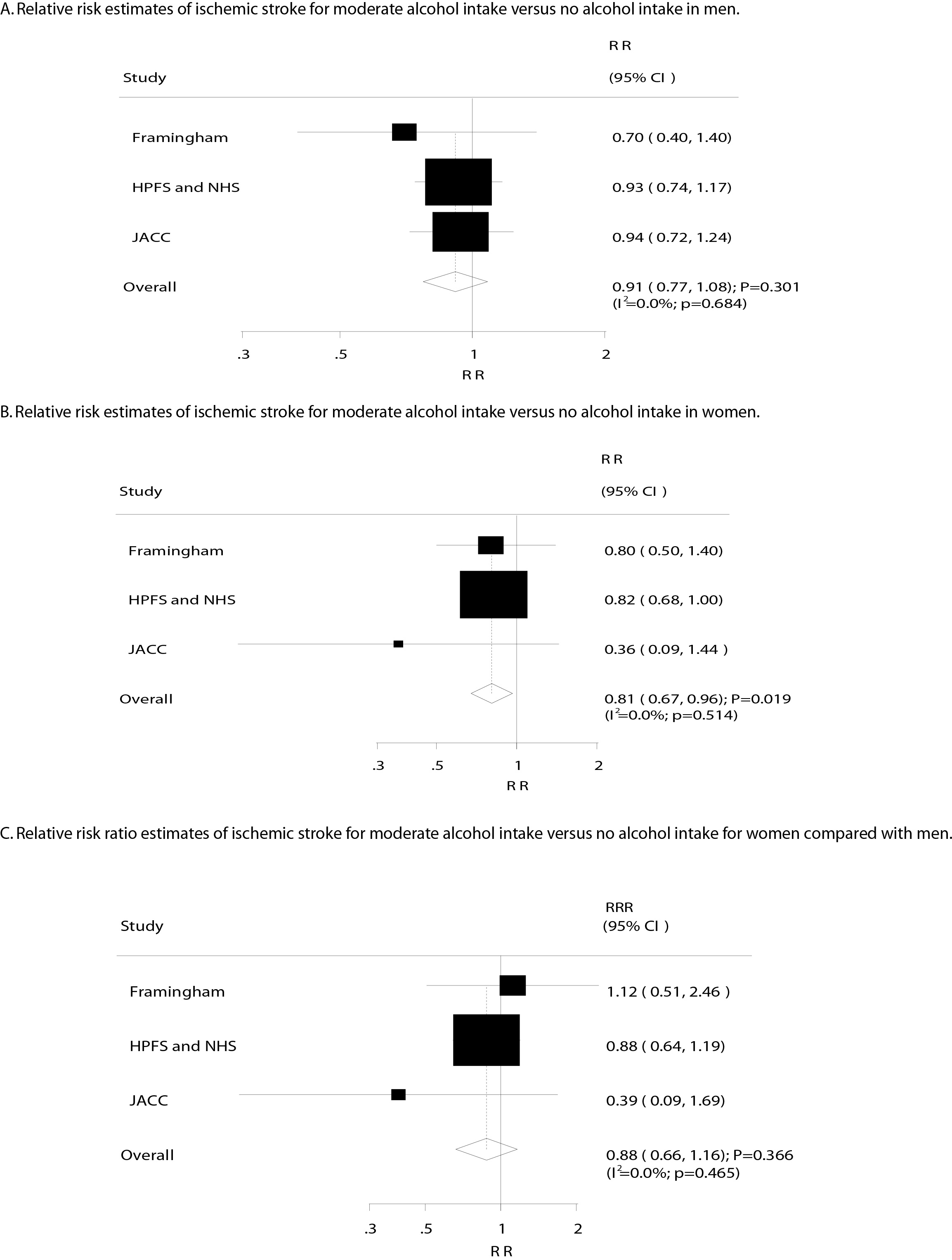


Figure S12. RR or RRR (female to male) of moderate alcohol intake and the risk of ischemic stroke.


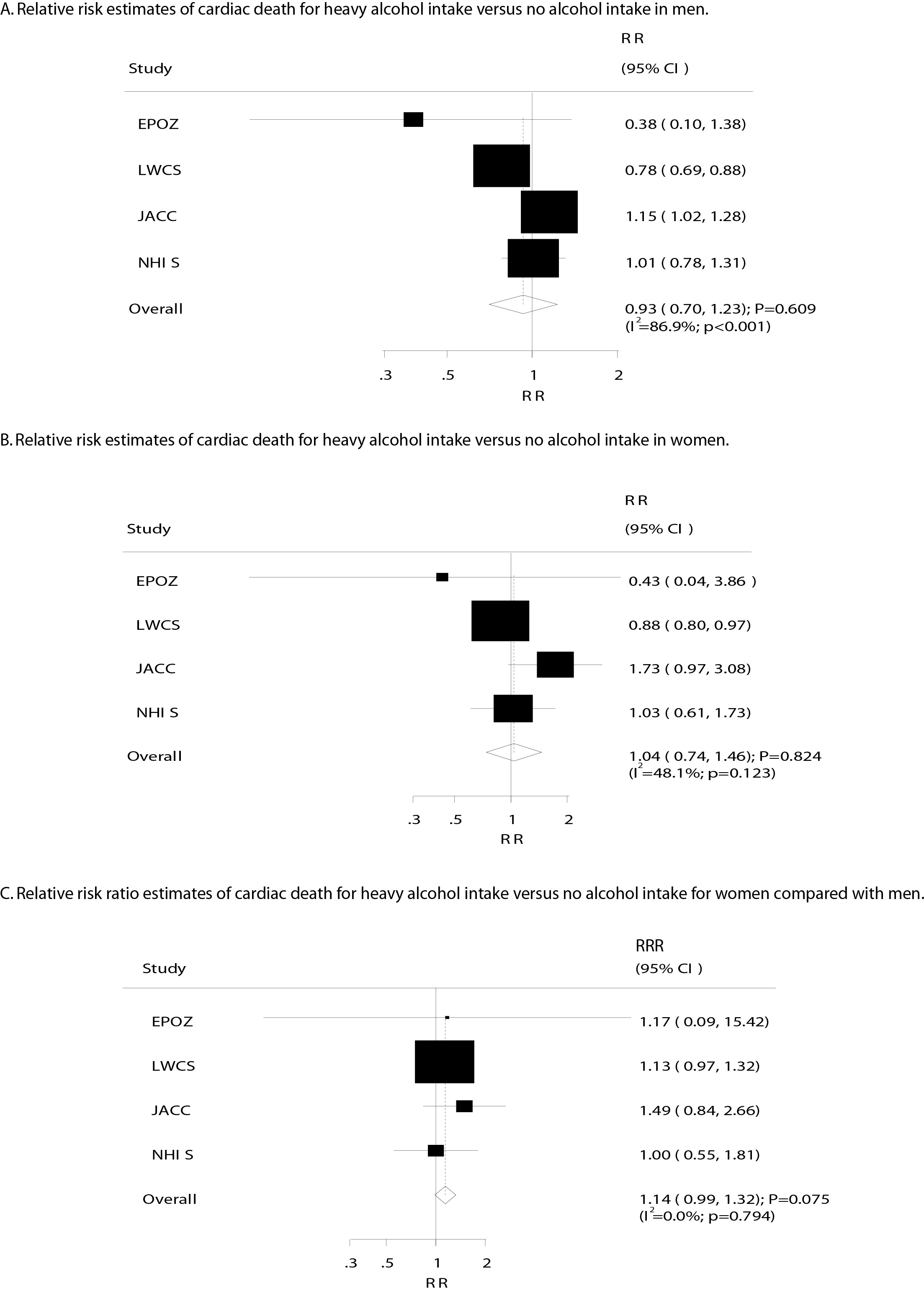


Figure S13. RR or RRR (female to male) of heavy alcohol intake and the risk of cardiac death.


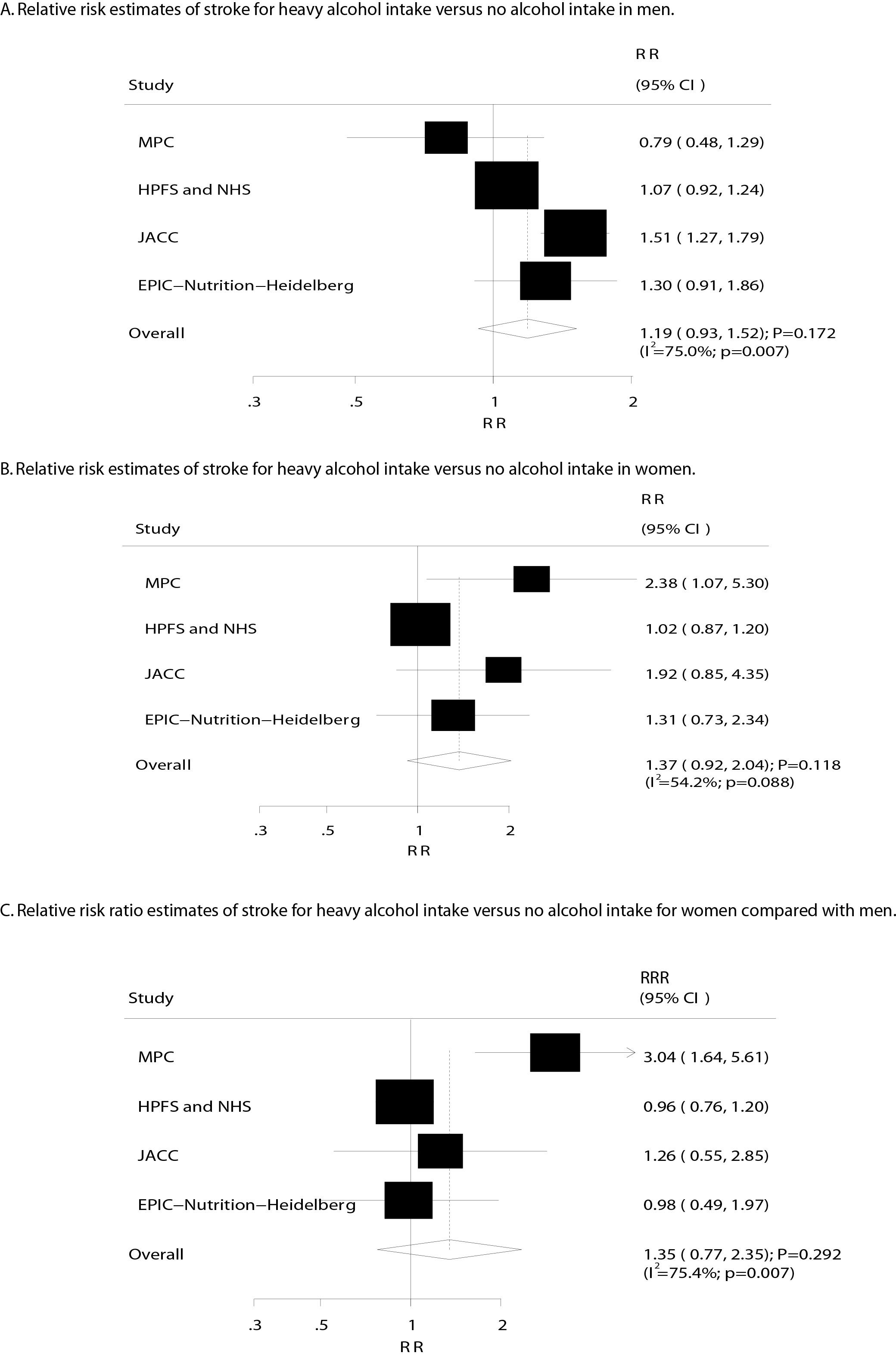


Figure S14. RR or RRR (female to male) of heavy alcohol intake and the risk of stroke.


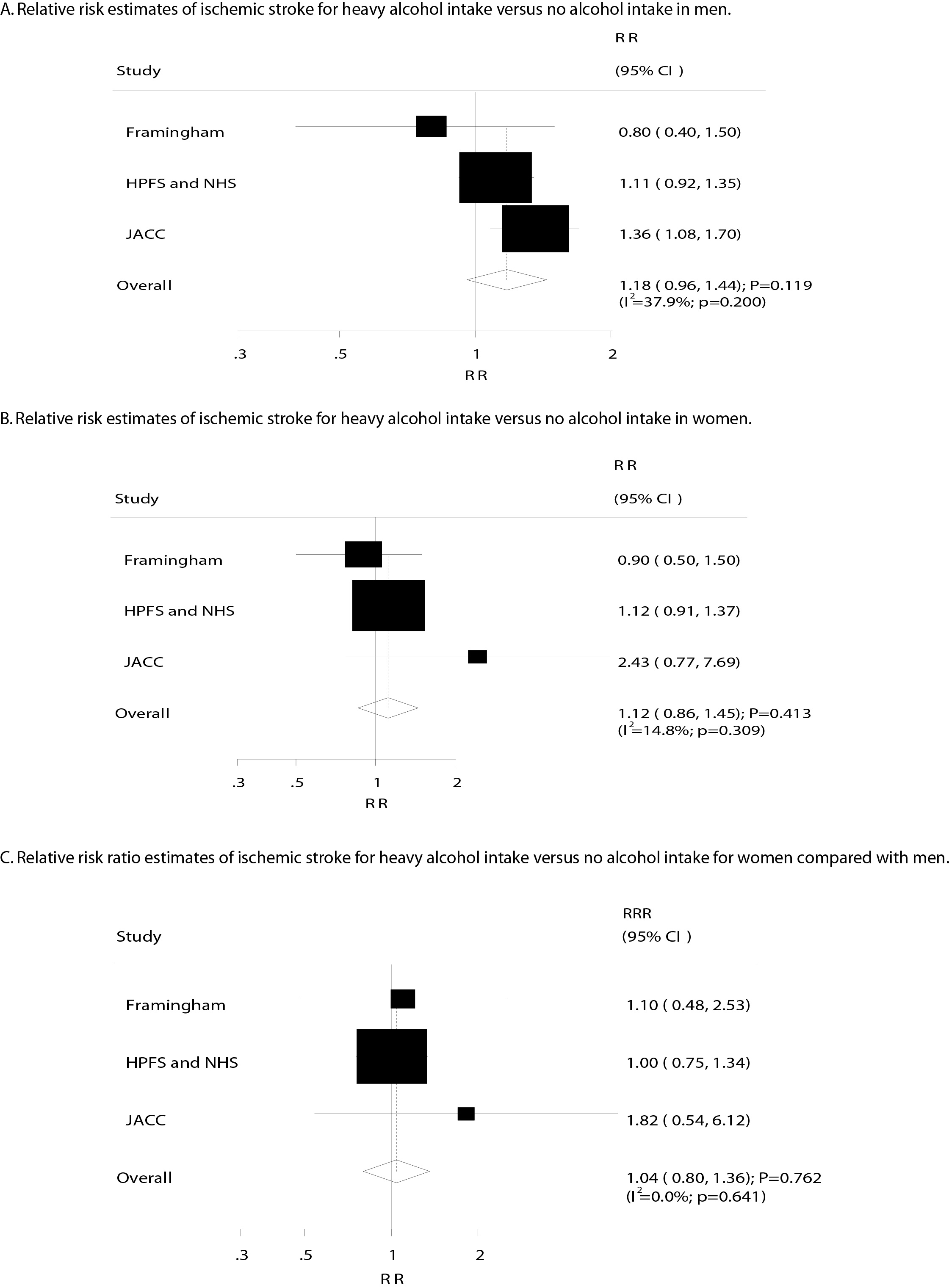


Figure S15. RR or RRR (female to male) of heavy alcohol intake and the risk of ischemic stroke.


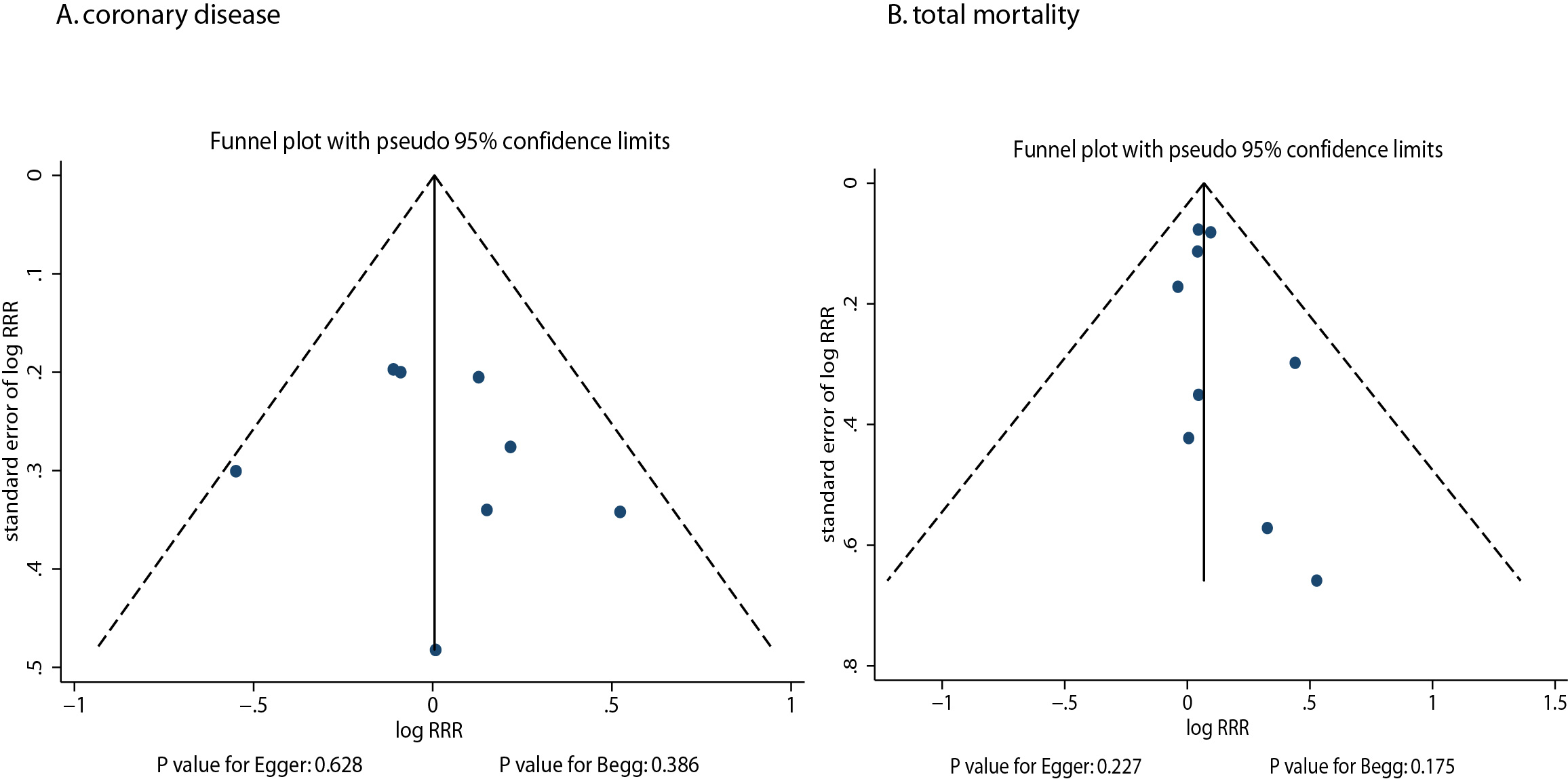


Figure S16. Funnel plot of RRR (female to male) for low alcohol intake.


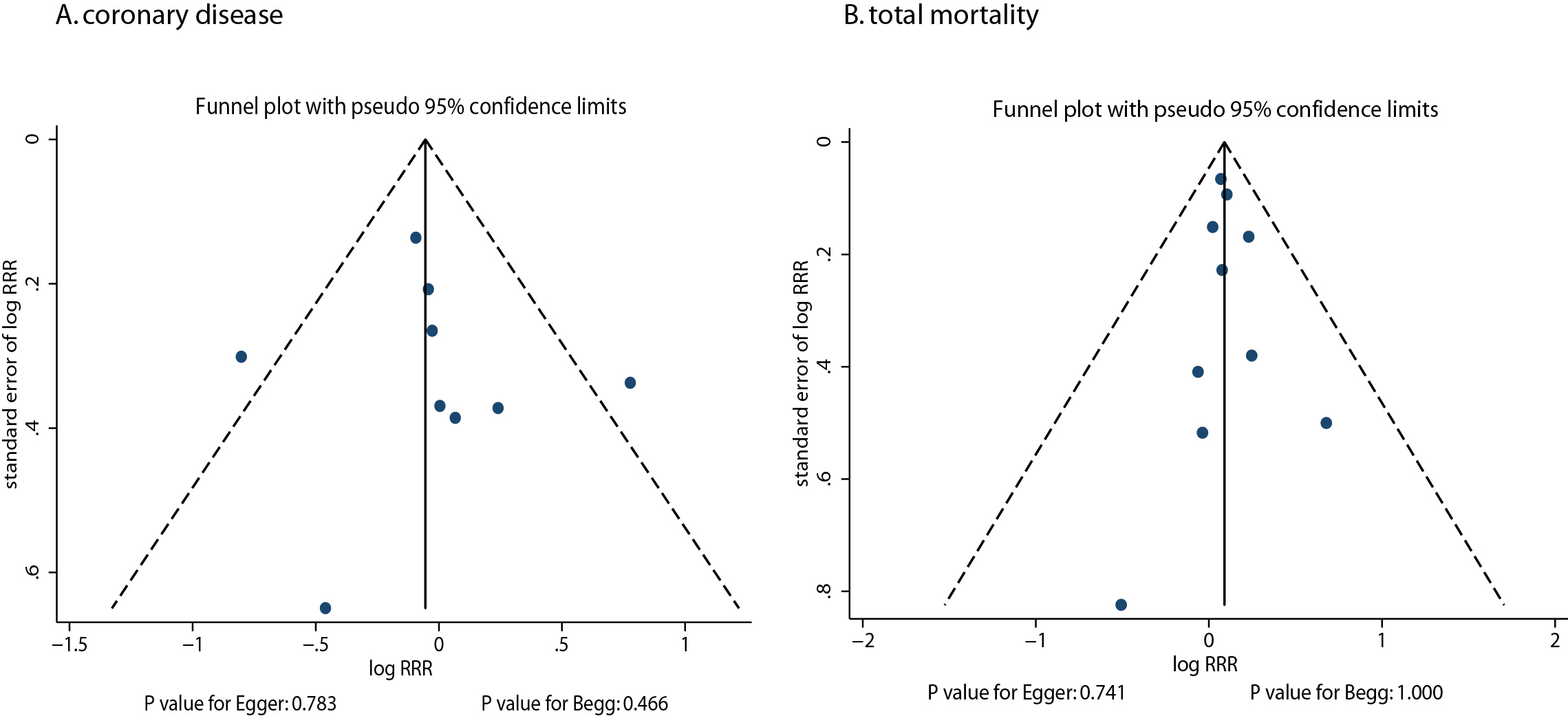


Figure S17. Funnel plot of RRR (female to male) for moderate alcohol intake.


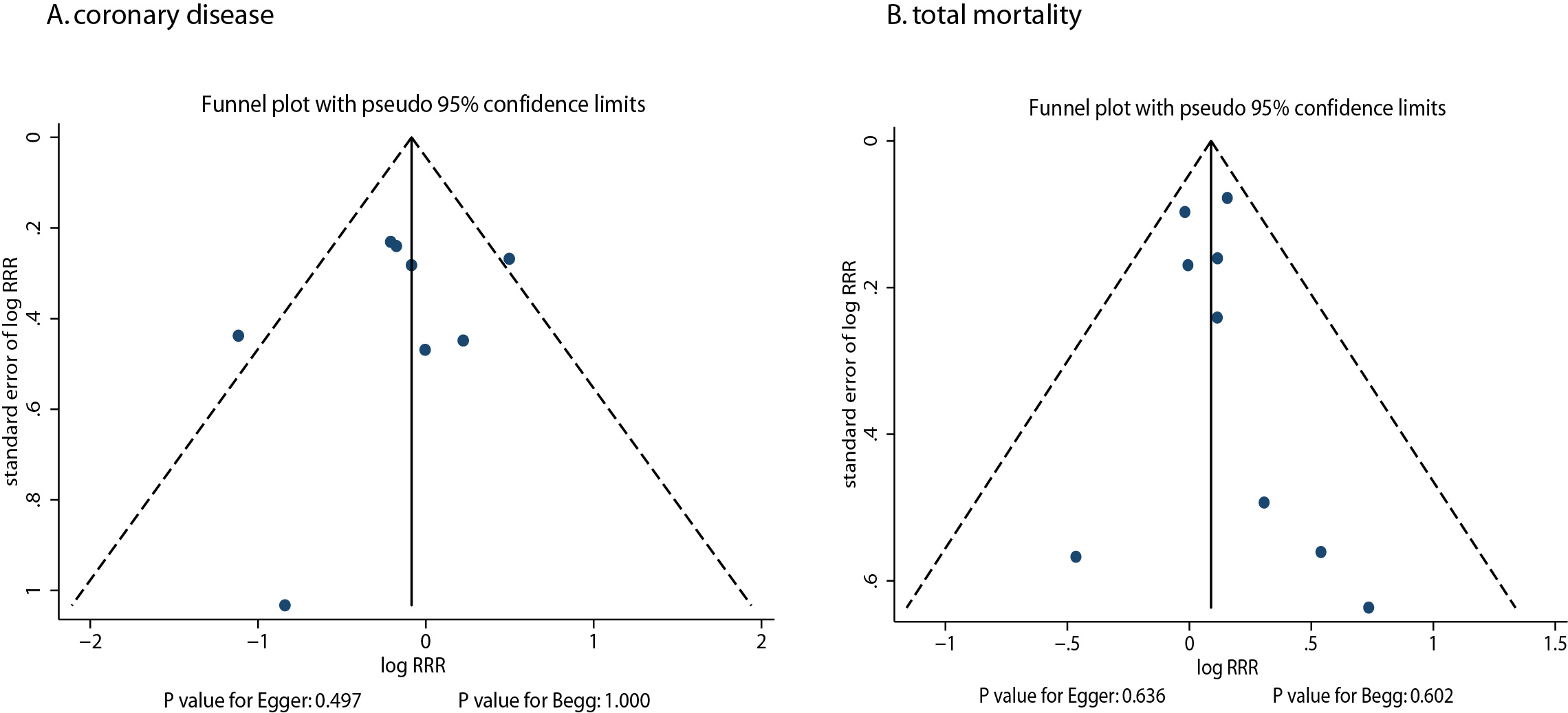


Figure S18. Funnel plot of RRR (female to male) for heavy alcohol intake.
